# A Testis‐Specific Aralkylamine *N*‐Acetyltransferase Regulates Dimorphic Sperm Function and Male Fertility in Moths

**DOI:** 10.1002/advs.202516374

**Published:** 2026-03-15

**Authors:** Hao Sun, Peng‐Yi Huang, Zhi‐Ruo Zhang, Cong‐Fen Gao, Subba Reddy Palli, Shun‐Fan Wu

**Affiliations:** ^1^ State Key Laboratory of Agricultural and Forestry Biosecurity College of Plant Protection Nanjing Agricultural University Nanjing China; ^2^ Department of Entomology University of Kentucky Lexington Kentucky USA

**Keywords:** arylalkylamine *N*‐acetyltransferase, dimorphic sperm, Lepidoptera, mitochondrial derivatives, sperm motility

## Abstract

Most Lepidopterans produce two distinct sperm types—nucleated eupyrene (fertile) and anucleate apyrene (non‐fertile), yet the genetic mechanisms governing this dimorphism remain poorly understood. Using the fall armyworm (*Spodoptera frugiperda*) as a model, we identified a Lepidoptera‐conserved, testis‐specific arylalkylamine *N*‐acetyltransferase (LTNAT) indispensable for male fertility. Functional analyses showed that LTNAT is expressed in both sperm types, and its depletion disrupts mitochondrial derivatives (MDs) in eupyrene sperm and drastically impairs apyrene sperm motility. Multi‐omics analyses revealed *LTNAT* mutation perturbs energy metabolism, lipid homeostasis, and transmembrane transport—alterations correlating with abnormal MD structure. Furthermore, *LTNAT* deficiency likely compromises apyrene sperm motility by downregulating *Sex‐lethal* and flagellar assembly genes (e.g., *dyneins* and *intraflagellar transport proteins*). CRISPR/Cas9‐mediated knockout of *LTNAT* homologs in two other lepidopteran species similarly reduced male fertility, confirming functional conservation. Mass release of *LTNAT*‐deficient males significantly suppressed female fertility in caged experiments. Collectively, *LTNAT* is a core regulator of dimorphic sperm development in Lepidoptera, highlighting its potential as a target for innovative insecticidal or genetic pest control.

## Graphical abstract

We identify a Lepidoptera‐conserved testis‐specific arylalkylamine *N*‐acetyltransferase (LTNAT) that governs male moth fertility via a novel mechanism. LTNAT loss disrupts eupyrene sperm mitochondrial derivatives and impairs apyrene sperm motility, offering a safe molecular target for innovative pesticides and genetic pest control.

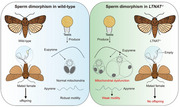

## 1 Introduction

Across the animal kingdom, sperm show striking morphological diversity and are among the most rapidly evolving cell types [1]. A single male can consistently produce two morphologically distinct sperm types within a common testis, a phenomenon known as sperm dimorphism [2]. Typically, only one sperm type can fertilize eggs and is referred to as the “fertile sperm” or eusperm, whereas the other, non‐fertilizing type is termed the “non‐fertilizing sperm” or parasperm. This kind of dimorphic sperm system has been independently reported in Mollusca [2], Annelida [3], Arthropoda [4, 5], and Chordata [6], suggesting that sperm dimorphism is a reproductive trait that has evolved repeatedly in different lineages.

In lepidopteran insects, dimorphic sperm is considered a remarkable characteristic of male reproduction. Except for two Micropterix species that lack typical apyrene sperm [7], nearly all lepidopteran males produce nucleated eupyrene sperm and anucleated apyrene sperm. In most examined species, apyrene sperm are thinner or shorter than eupyrene sperm [8]. Although the two sperm types originate from the same germ‐cell lineage, they differ markedly in their production timing and the meiotic program they undergo [4, 9]. Eupyrene spermatogenesis usually begins during the larval stage and ceases after pupation, whereas apyrene spermatogenesis is initiated only around pupation [4, 9]. Cytological and physiological studies have shown that the switch from producing eupyrene sperm to producing apyrene sperm is regulated by a macromolecular factor in the hemolymph, whose accessibility to the testes changes with developmental stage and thereby enforces organism‐wide control over dichotomous spermatogenesis [4, 9]. Eupyrene spermatogenesis involves a complete meiotic program accompanied by nuclear protein remodeling [4, 9]. By contrast, meiosis in apyrene sperm cells is highly abnormal: chromosomes fail to form a normal metaphase plate, and multiple micronuclei arise at anaphase [4, 9]. These micronuclei then move toward the posterior end of the sperm bundle and are ultimately extruded, producing completely anucleate sperm cells [4, 9].

Although apyrene sperm do not fertilize eggs, they play key physiological roles in the reproductive process of lepidopteran insects [10, 11, 12]. Genetic studies in the silkworm *Bombyx mori* have shown that mutation of *Sex‐lethal* (*Sxl*) disrupts apyrene sperm motility and thereby blocks efficient migration of eupyrene sperm from the bursa copulatrix to the spermatheca [10]. In the polyandrous butterfly *Pieris napi*, apyrene sperm can delay female remating, thus potentially reducing the risk of the male's gametes being displaced in subsequent sperm competition [11]. Studies of spermatogenesis in the lepidopteran model *B. mori* have yielded important insights: knockout of *Maelstrom* (*Mael*) [13], *protein arginine methyltransferase 5* (*Prmt5*), or *Vasa* [14] causes developmental defects in both sperm types. In addition, several genes, including *Polyamine modulated factor 1 binding protein* (*PMFBP1*) [15], *poly(A)‐specific ribonuclease‐like domain containing 1* (*PNLDC1*) [10], *Hua enhancer 1* (*Hen1*) [16], *Pizeo* [17], are specifically required for eupyrene spermatogenesis and transfer, indicating that dimorphic sperm are controlled by distinct genetic regulatory modules. Despite these advances, our understanding of the genetic basis that regulates dimorphic sperm development in Lepidoptera remains limited. Moreover, many highly fecund lepidopteran pests pose serious threats to global food production [18]. Thus, identifying the key genes involved in spermatogenesis also provides molecular targets for pest genetic control.

The fall armyworm *Spodoptera frugiperda* is a lepidopteran pest that produces dimorphic sperm and has evolved resistance to multiple chemical insecticides [19, 20]. Here, we use *S. frugiperda* as a model to investigate the molecular basis of spermatogenesis. Using RNA‐seq and CRISPR/Cas9 approaches, we identified a male sterility gene *LOC118263478*. Structural prediction, catalytic activity assays, and phylogenetic analyses together indicated that *LOC118263478* encodes a Lepidoptera‐ conserved testis‐specific arylalkylamine *N*‐acetyltransferase, which we named LTNAT. LTNAT is strongly conserved in amino acid sequence and function among lepidopteran insects. Mechanistic analyses showed that LTNAT is expressed in both sperm types and regulates the formation of mitochondrial derivatives in eupyrene sperm as well as the motility of apyrene sperm. Finally, integrated transcriptomic and proteomic analyses were performed to explore the potential pathways by which LTNAT regulates dimorphic sperm function. These results provide new molecular insights into dimorphic sperm development and broaden the functional repertoire of insect arylalkylamine *N*‐acetyltransferases.

## 2 Results

### 2.1 Comparative Analysis of Testis Transcriptome to Screen Testis‐Specific Genes

**FIGURE 1:**
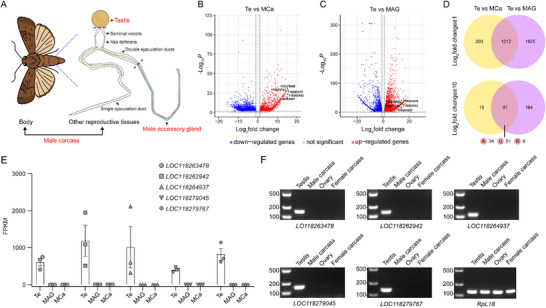
Identification of five testis‐specific genes through comparative transcriptome analysis and RT‐PCR. (A) Schematic diagram of collecting Te, MAG, and MCa for transcriptome sequencing. (B) Volcano plot shows DEGs in the Te vs MCa. (C) Volcano plot analysis of DEGs in the Te vs MAG group. Upregulated, downregulated, and not significant genes are marked with red, blue, and gray, respectively. Locations of the five candidate genes are marked. (D) The Venn diagram illustrates genes commonly upregulated in the Te vs both MCa and MAG. Gene categories: A, annotated genes; U, uncharacterized genes; N, novel genes. (E) The FPKM expression values of *LOC118263478*, *LOC118262942*, *LOC118264937*, *LOC118279045*, and *LOC118279767* in the Te, MAG, and MCa. (F) RT‐PCR validation of testis‐specific expression patterns. *RpL18* served as the internal reference control.

To explore the molecular basis of dichotomous spermatogenesis, we conducted transcriptome sequencing on testis (Te), male accessory gland (MAG), and male carcass (MCa) tissues from 1‐day‐old adult males (Figure 1A). Sample correlation and principal component analysis (PCA) showed low pairwise correlations (<0.6) and distinct clustering among the three tissues, indicating significant gene expression differences between the Te and the other tissues (Figure S1, Supporting Information). Volcano plots illustrate the differentially expressed genes (DEGs) between Te versus (vs) MCa or MAG (Figure 1B,C). Specifically, Te vs MCa identified 1416 significantly upregulated and 2040 significantly downregulated DEGs (Figure 1B). Similarly, Te vs MAG revealed a large number of DEGs, including 3137 significantly upregulated genes (Figure 1C). Using a fold‐change (FC) threshold of ≥2, we found 1212 genes commonly upregulated in both Te comparisons (Figure 1D). Even at a stringent fold‐change threshold of 1024 (|log_2_FC|≥10), 91 genes remained commonly upregulated (Figure 1D). Among these, 51 lack functional annotation, 30 have descriptions, and 6 are novel genes (Figure 1D). Notably, more than half of the commonly upregulated high‐confidence DEGs lack functional annotation, which limits our understanding of spermatogenesis.

To identify regulatory pathways linked to testis‐enriched gene expression, we performed Kyoto Encyclopedia of Genes and Genomes (KEGG) and Gene Ontology (GO) enrichment analyses on genes upregulated in the Te. KEGG analysis showed enrichment in various metabolic pathways, phagosome formation, ABC transporters, ECM‐receptor interactions, amino acid and cofactor biosynthesis, along with MAPK and Wnt signaling pathways (Figure S2A,B, Supporting Information). Likewise, GO analysis indicated enrichment in processes related to cell communication, signal transduction, development, and extracellular region (Figure S2C,D, Supporting Information).

The MAG is an important part of the male reproductive system, filled with various reproductive genes related to mating, spermatophore formation, and sperm activation [21, 22]. MCa, as a non‐reproductive somatic tissue throughout the body, is often used as a background for screening germline genes [23, 24]. From this perspective, we believe that genes truly strongly related to spermatogenesis itself may be preferentially obtained from Te vs MCa. Therefore, we selected the top five upregulated uncharacterized genes from the 91 DEGs ranked by the Te vs MCa comparison for functional exploration: *LOC118263478*, *LOC118262942*, *LOC118264937*, *LOC118279045* (annotated as galectin‐4‐like), and *LOC118279767* (annotated as chorion class A proteins Ld9‐like) (Figure 1B–D). Transcriptome quantitative data showed that these five genes are exclusively expressed in Te (Figure 1E). Further, RT‐PCR analysis validated this result (Figure 1F).

### 2.2 CRISPR/Cas9‐Mediated Knockout of *LOC118263478* Leads to Male Sterility

**FIGURE 2:**
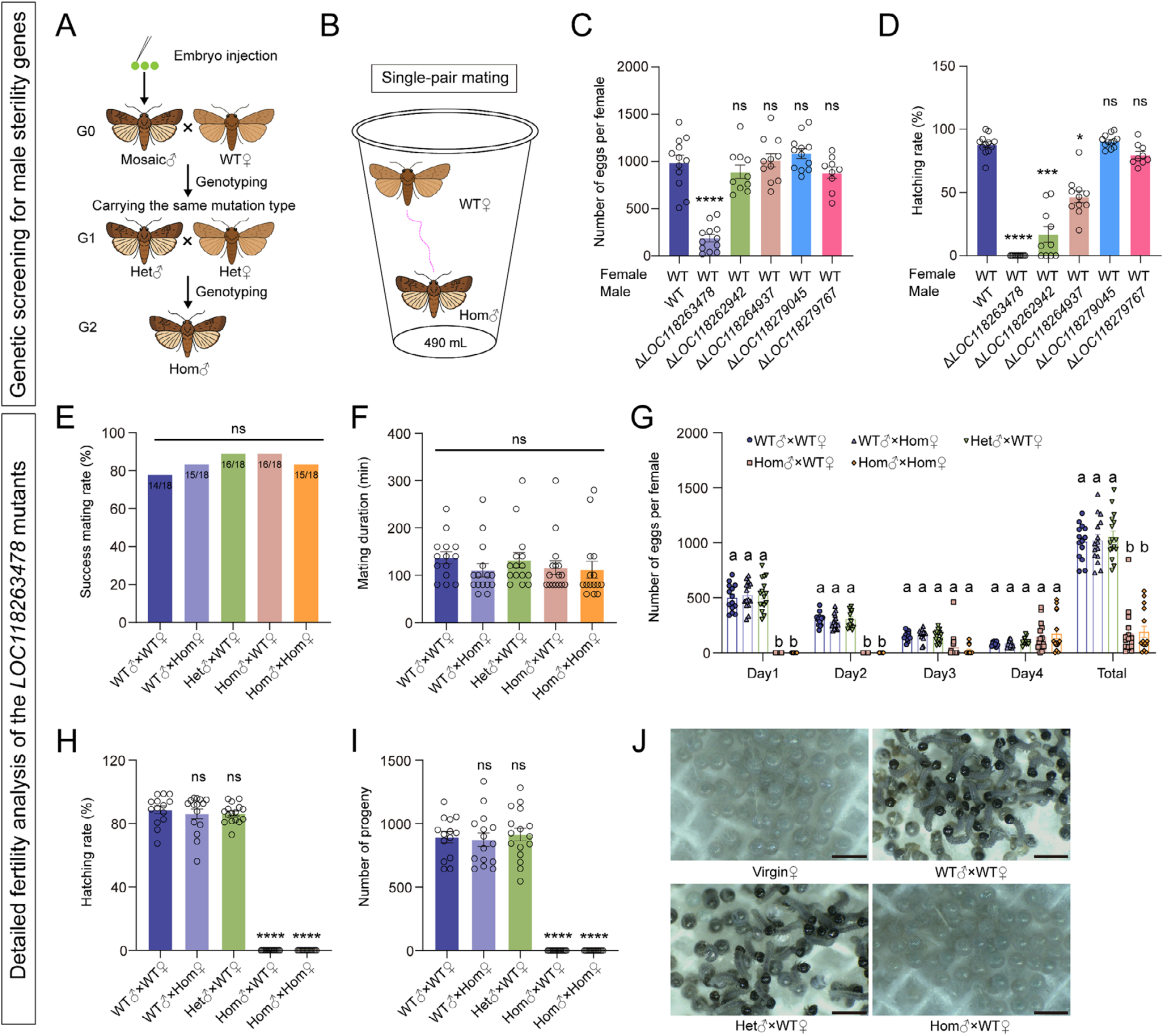
Knockout of *LOC118263478* causes male sterility. (A) Flow chart for constructing gene mutants. Het, heterozygous mutant; Hom, homozygous mutant. (B) Schematic of the fertility assay. A moth pair was placed in a 490‐mL plastic cup for mating. Mating status was observed manually under red light. (C) Number of eggs laid per female. *n* = 9–12 pairs. Compare the mean of each column with the mean of the control group WT × WT combination. One‐way ANOVA followed by Tukey's multiple comparisons test; ns, not significant; ****p < 0.0001. (D) Egg hatching rate (%). *n* = 9–12. Kruskal–Wallis test with Dunn's multiple comparisons test; ns, not significant; *p < 0.05; ***p < 0.001; ****p < 0.0001. (E) Mating success rate (successful matings/total pairs). Fisher's exact test; ns, not significant. (F) Mating duration. Mating status was checked every 15 min under red light. *n* = 14–16 pairs. Kruskal–Wallis test with Dunn's multiple comparisons test; ns, not significant. (G) Daily and total egg production per female. *n* = 14–16 pairs. Kruskal–Wallis test with Dunn's multiple comparisons test. Different lowercase letters indicate significant differences (*p* < 0.01). (H) Egg hatching rate (%). *n* = 14–16. Compare the mean of each column with the mean of the control group WT × WT combination. Kruskal–Wallis test with Dunn's multiple comparisons test; ns, not significant; ****p < 0.0001. (I) Number of hatched larvae. *n* = 14–16. Compare the mean of each column with the mean of the control group WT × WT combination. Kruskal–Wallis test with Dunn's multiple comparisons test; ns, not significant; ****p < 0.0001. (J) Egg hatching status 72 h (h) post‐spawning for indicated crosses. Scale bar: 1 mm. Genotypes: Hom, *LOC118263478* homozygous mutant; Het, *LOC118263478* heterozygous mutant; WT: wild‐type. All data presented as mean ± SEM.

To examine the potential roles of five testis‐specific genes in male fertility, we used CRISPR/Cas9 technology to generate gene knockout mutants. A mixture of Cas9 protein and two single guide RNAs (sgRNAs) was injected into fresh embryos, followed by multi‐generation insect rearing and genetic crosses to obtain homozygous mutants for each gene (Figure 2A). Details on the number of injected eggs, hatching rate, pupation rate, and G0 mutation rate are provided in Table S2 (Supporting Information). We successfully generated the *LOC118263478* mutant with a 92 base pairs (bp) deletion, the *LOC118262942* mutant with a 56 bp deletion, the *LOC118264937* mutant with a 4 bp insertion, the *LOC118279045* mutant with a 64 bp deletion, and the *LOC118279767* mutant with a 16 bp deletion (Figures S3–S7, Supporting Information). Direct sequencing of PCR products from 20 individuals per line confirmed the presence of the expected indels (Figures S3–S7, Supporting Information). Each mutation caused premature termination of protein translation (Figures S3–S7, Supporting Information).

To evaluate the effect of single‐gene knockout on male fertility, we conducted single‐pair mating assays (Figure 2B). While females mated with *LOC118262942* or *LOC118264937* mutant males laid a normal number of eggs, the egg hatching rate was significantly lower than in the control group (Figure 2C,D; Figure S8, Supporting Information). We also found that mutation of *LOC118263478* severely decreased male fertility, as indicated by the significantly reduced egg production by their female partners (Figure 2C). Remarkably, the hatching rate of eggs laid by wild‐type (WT) females after mating with *LOC118263478* mutant males was 0 (Figure 2D). Conversely, the number of eggs and hatching rate from crosses using *LOC118279045* or *LOC118279767* mutant males with WT females were similar to controls (Figure 2C,D). These findings demonstrate that knockout of *LOC118263478* causes complete male sterility, while knockout of *LOC118262942* and *LOC118264937* considerably diminishes male fertility.

Knockout of *LOC118263478* resulted in a fully penetrant, complete male‐sterile phenotype, indicating that this gene is indispensable for male reproductive success. Therefore, we prioritized *LOC118263478* for subsequent mechanistic analyses. We comprehensively assessed the impact of *LOC118263478* disruption on the mating and egg‐laying behaviors in both sexes. Analysis of mating behavior showed that the mutation of *LOC118263478* did not affect the mating rate and mating duration (Figure 2E,F). The number of offspring produced by the mating of homozygous mutant females with WT males was not significantly different from that of the control group (Figure 2G–I). Since *LOC118263478* is only expressed in the male testis, this result is predictable. In addition, *LOC118263478* acts in a recessive manner, and its heterozygous mutation does not impair male fertility (Figure 2G–I). Interestingly, we found that mating with homozygous mutant males significantly reduced the egg‐laying number of females, especially during the first two days post‐mating when females failed to lay eggs (Figure 2G). Finally, the eggs laid by WT females mated with homozygous mutant males also did not hatch (Figure 2J). Considering the sterility of *LOC118263478* homozygous males, we crossed heterozygous males with homozygous females to obtain homozygous offspring. Due to the 92‐bp deletion, we easily identified the homozygous mutants by the PCR band pattern (Figure S9, Supporting Information).

### 2.3 Biochemical Characterization of LOC118263478 With Catalytic Activity Toward Polyamines and Aralkylamines

**FIGURE 3:**
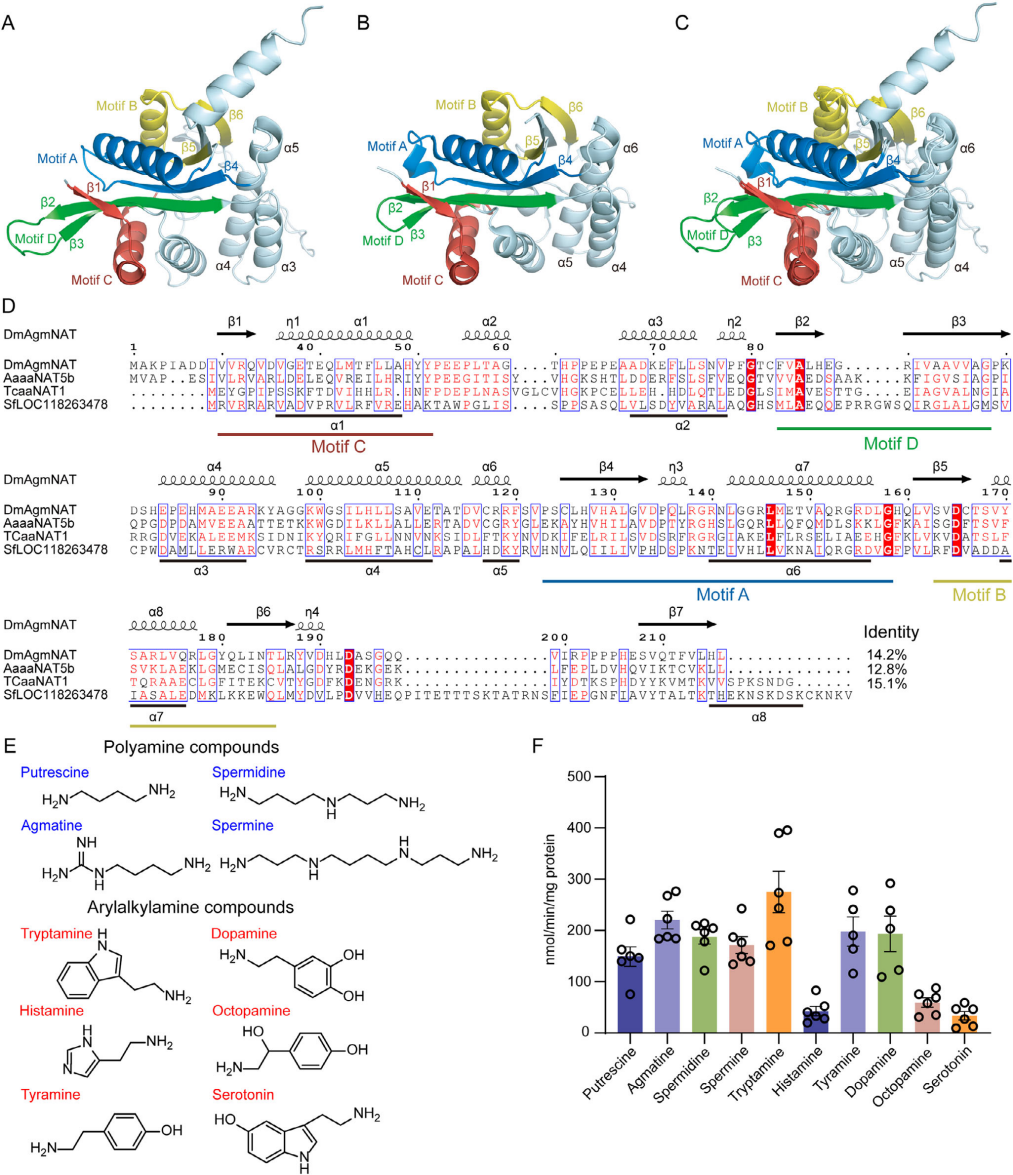
*LOC118263478* encodes an arylalkylamine *N*‐acetyltransferase. (A) AlphaFold predicts the structure of LOC118263478. Motif C (including β1), Motif D (including β2 and β), Motif A (including β4), and Motif B (including β5 and β6) are shown in red, green, blue, and yellow, respectively. Other regions of the structure are in gray. Three α‐helices between β3 and β4 are marked. (B) The structure of DmagmNAT (PDB: 5k9n). Label the structure in the same way as in A. (C) Compare the structure of LOC118263478 with that of DmagmNAT. Label the structure in the same way as in A. (D) Protein sequence alignment of LOC118263478, DmagmNAT, AaaaNAT5b, and TcaaNAT1. The secondary structure of DmagmNAT is labeled above the sequence. Completely identical residues are marked with white letters on a red background. Residues that are not completely identical in sequence but have similar physicochemical properties are marked with red letters on a white background. The α‐helix structure of LOC118263478 is labeled below the alignment. Motifs C, D, A, and B are located on the red, green, blue, and yellow lines, respectively. (E) Four polyamine compounds and six arylalkylamine compounds were used for the DTNB colorimetric assay. (F) The activity of LOC118263478 was assessed against four polyamine compounds and six arylalkylamine compounds.

To understand the relationship between LOC118263478 and male fertility, we first conducted bioinformatics analysis to obtain the structural information of this protein. LOC118263478 lacks a signal peptide and transmembrane domains, and SMART and NCBI CD‐search did not detect any annotated conserved domains. AlphaFold prediction revealed that the LOC118263478 protein consists of 7 β‐sheets and 8 α‐helices, with its central region composed of antiparallel β‐sheets (Figure 3A). Structural comparison using the AlphaFold Protein Structure Database showed that LOC118263478 is most similar to *Drosophila melanogaster* agmatine *N*‐acetyltransferase (DmagmNAT) (Figure 3B,C). From the N‐ to the C‐terminus, LOC118263478 contains four sequential conserved motifs: motif C, motif D, motif A, and motif B (Figure 3D). As in other insect aaNATs, the LOC118263478 has a loop‐helix‐turn‐helix structure between β3 and β4 (Figure 3A). Despite these structural similarities, LOC118263478 shares only 14.2%, 12.8%, and 15.1% sequence identity with DmagmNAT, *Aedes aegypti* aralkylamine *N*‐acetyltransferase 5b (AaaaNATb), and *Tribolium castaneum* aralkylamine *N*‐acetyltransferase (TcaaNAT), respectively (Figure 3D). Different from the α‐helix distribution of the other three proteins, the α‐helix 8 of LOC118263478 is located at the C‐terminus (Figure 3D). DTNB assays of the Ni‐NTA affinity‐enriched recombinant LOC118263478 protein indicated measurable catalytic activity toward four polyamines and three aralkylamine substrates (Figure 3E,F; Figure S10, Supporting Information).

### 2.4 The Function of LOC118263478 (LTNAT) in Regulating Male Fertility Is Conserved in Moths

**FIGURE 4:**
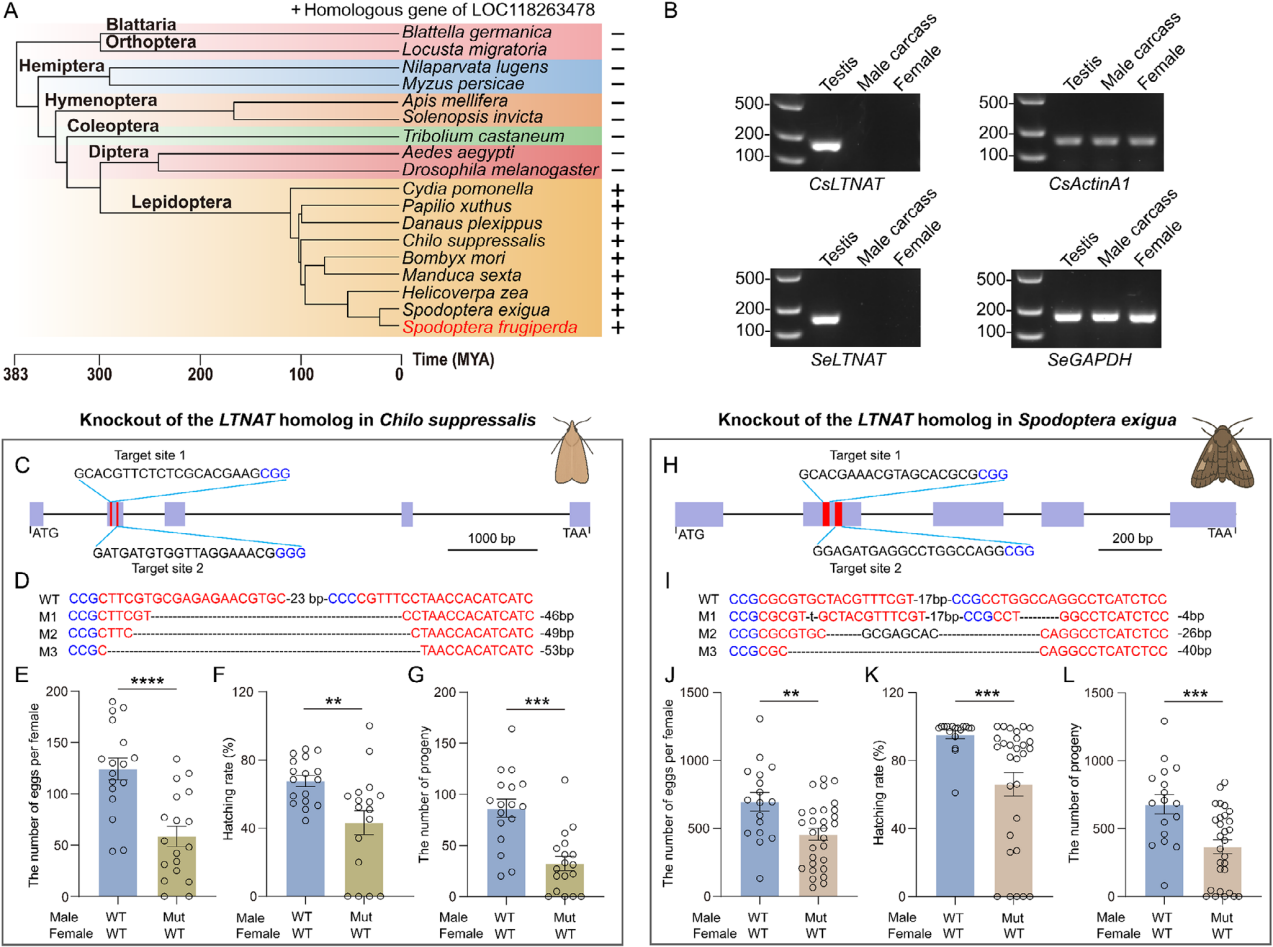
The function of LTNAT is conserved in moths. (A) Distribution of LTNAT homologs across insect species. A plus sign (+) indicates the presence of LTNAT homologous genes, while a minus sign (−) indicates the absence of *LTNAT* homologous genes. (B) *LTNAT* homologs exhibit a testis‐specific expression pattern in the two moths. RT‐PCR analysis of the expression patterns of *LTNAT* in *Chilo suppressalis* (*CsLTNAT*) and *Spodoptera exigua* (*SeLTNAT*). *CsActionA1* and *SeGAPDH* serve as housekeeping genes. (C) CRISPR/Cas9‐mediated knockout of *CsLTNAT*. The sgRNA position is indicated by a red box; the PAM sequence is marked in blue. Scale bar, 1000 bp. (D) Sequence analysis of *CsLTNAT* mutations in the G0 generation. The WT sequence is shown on top; mutant sequences are shown below. Deleted bases are indicated by dashed lines; deletion size (number of bases, −) is indicated on the right. sgRNA and PAM sites are highlighted in red and blue, respectively. (E) Number of eggs laid. *n* = 17–18. Student's *t* test; ns, ****p < 0.0001. (F) Egg hatchability. *n* = 17–18. Student's *t* test; **p < 0.01. (G) Number of hatched larvae. *n* = 17–18. Mann–Whitney *U* test; ***p < 0.001. (H) CRISPR/Cas9‐mediated knockout of *SeLTNAT*. Positions of the two designed sgRNAs are indicated by red boxes. sgRNA sequences are shown in black; PAM sites are in blue. Scale bar, 1000 bp. (I) Detection of mutation types in CRISPR/Cas9‐edited *SeLTNAT* G0 generation. The WT sequence is shown at the top. Target sites are marked in red; PAM sequences are in blue. Dashed lines indicate deletions; deletion size (number of bases, −) is shown on the right. (J) Number of eggs per female. *n* = 17–29. Student's *t* test; ns, **p < 0.01. (K) Egg hatching rate. *n* = 17–29. Mann–Whitney *U* test; ***p < 0.001. (L) Number of progeny. *n* = 17–29. Mann–Whitney *U* test; ***p < 0.001.

tBLASTn and BLASTp analyses revealed that LOC118263478 homologs are widely distributed in lepidopteran insects, but absent from representative species of other insect orders (Figure 4A). Multiple sequence alignment showed that LOC118263478 homologs are highly conserved among Lepidoptera, particularly across four key motifs (Figure S11, Supporting Information). Phylogenetic analysis of 65 LOC118263478 homologs from 15 lepidopteran subfamilies indicated that homologs from the same subfamily consistently formed well‐supported monophyletic clades, suggesting that this gene has primarily evolved in concert with lineage diversification (Figure S12, Supporting Information). RT‐PCR confirmed a testis‐specific expression pattern of LOC118263478 homologs in two other moths (Figure 4B). To clarify the evolutionary relationships between LOC118263478 homologs and aaNATs in mammals and insects, we constructed a phylogenetic tree (Figure S13, Supporting Information). LOC118263478 grouped with lepidopteran homologs to form a monophyletic clade distinct from other aaNAT lineages, suggesting that it represents a lineage‐diverged aaNAT subfamily in Lepidoptera, hereafter referred to as “lepidopteran type aaNATs” (Figure S13, Supporting Information). Based on the above results, we preliminarily named the *LOC118263478* a *Lepidoptera‐conserved testis‐specific aaNAT* (*LTNAT*).

To assess the functional conservation of LTNAT in male fertility, we knocked out *LTNAT* homologs in *Chilo suppressalis* and *Spodoptera exigua* using CRISPR/Cas9. We employed multiple sgRNAs targeting adjacent sites in exon 2 to produce G0 mosaicism (Figure 4C,H). Subcloning and sequencing of PCR products confirmed mutations in G0 mutants (Figure 4D,I). Single‐pair mating assays indicated that, compared with the control group, G0 mutant males significantly reduced the egg production, egg hatching rate, and the number of viable offspring of their female mates (Figure 4E–G,J–L). Based on the above results, we speculate that the function of LTNAT in regulating male fertility is conserved in moths.

### 2.5 *LTNAT* Regulates Eupyrene Spermatogenesis and Apyrene Sperm Motility

**FIGURE 5:**
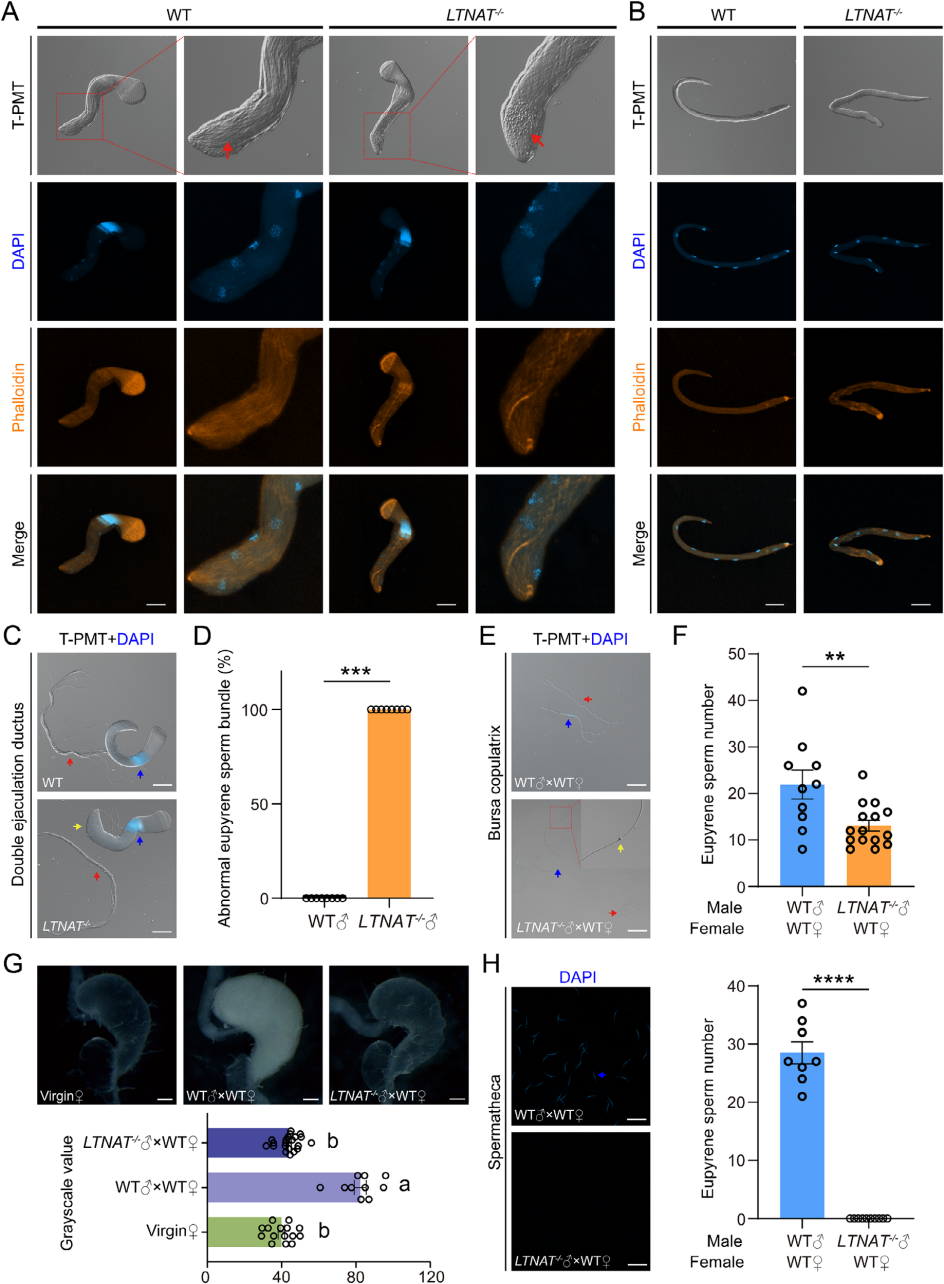
*LTNAT* deficiency disrupts eupyrene spermatogenesis and apyrene sperm motility. (A) Fluorescence staining of eupyrene sperm bundles in testes from WT and *LTNAT^−/−^* adult males. Scale bar: 20 µm. The image on the right is a 6‐fold local magnification of the image on the left. The red arrow points to the tail of the eupyrene sperm bundle. Nuclei (blue) stained with DAPI; filamentous actin (red) stained with TRITC‐phalloidin. (B) Fluorescence staining of apyrene sperm bundles in testes from WT and *LTNAT^−/−^* adult males. Scale bar: 20 µm. Nuclei (blue) stained with DAPI; filamentous actin (red) stained with TRITC phalloidin. (C) Sperm staining within the duplex ejaculatory ducts of WT and *LTNAT^−/−^* males. Scale bar, 20 µm. The blue arrow points to the eupyrene sperm bundle, the red arrow points to the apyrene sperm, and the yellow arrow points to the punctate protrusions. (D) The proportion of abnormal eupyrene sperm bundles in the testes and double ejaculatory ducts. *n* = 8. Mann–Whitney *U* test; ***p < 0.001. (E) Sperm staining in the bursa copulatrix of females 1 h post‐mating. Scale bar: 20 µm. The blue arrow, red arrow, and yellow arrow point to eupyrene sperm, apyrene sperm, and punctate protrusions, respectively. (F) Count the number of eupyrene sperm in the spermatophore. *n* = 10–15. Student's *t*‐test; **p < 0.01. (G) Grayscale analysis of spermathecae from females 24 h post‐mating. One‐way ANOVA followed by Tukey's multiple comparisons test. Different letters indicate significant differences, *p* < 0.05. Scale bar: 200 µm. (H) Sperm staining in the spermathecae of females 24 h post‐mating. The blue arrow represents eupyrene sperm. Scale bar: 20 µm. (I) Count the number of eupyrene sperm in the spermathecae of females 24 h post‐mating. *n* = 8. Mann–Whitney *U* test; ****p < 0.001.

Male sterility in lepidopteran insects is often caused by defects in the development or transfer of dimorphic sperm [10, 15]. Thus, to understand why loss of *LTNAT* leads to male sterility, we examined sperm development in *LTNAT^−/−^* males and the transport of *LTNAT^−/−−^* male sperm in the male and female reproductive tracts. Fluorescent staining of the sperm of 1‐day‐old adults showed that *LTNAT* disruption did not affect the position and arrangement of nuclei within eupyrene sperm bundles (Figure 5A). However, unlike the smooth tails observed in WT, the tails of eupyrene sperm bundles in *LTNAT^−/−−^* males displayed punctate swellings (Figure 5A). By contrast, the morphology of apyrene sperm bundles was indistinguishable between WT and *LTNAT^−/−−^* males (Figure 5B).

We next assessed sperm transfer within the male reproductive tract. Imaging sperm in the duplex ejaculatory ducts of WT and *LTNAT^−/−^* males showed that apyrene sperm bundles were dissociated, whereas eupyrene sperm remained bundled and exhibited punctate protrusions at their tails (Figure 5C). The results indicated that the initial transfer of sperm into the duplex ejaculatory ducts was normal. We then counted the proportion of abnormal eupyrene sperm bundles in the testes and duplex ejaculatory ducts of *LTNAT^−/−^*. We found that all eupyrene sperm bundles in *LTNAT^−/−^* were abnormal (Figure 5D). To pinpoint when these punctate swellings arise, we stained sperm bundles in testes from L6D4 larvae to follow the development process of eupyrene sperm. At the early elongation stage of eupyrene sperm bundles in *LTNAT^−/−^*, no punctate swellings were observed (Figure S14, Supporting Information). Thus, we inferred that swelling occurs during the final elongation stage when eupyrene sperm bundles mature.

During mating, sperm and seminal fluid are transferred to the bursa copulatrix, where eupyrene sperm bundles dissociate, and apyrene sperm become motile [10]. Motile apyrene sperm then assist eupyrene sperm in moving from the bursa copulatrix to the spermatheca [10]. To assess this process, we stained and counted sperm in the bursa copulatrix 1 h after mating with *LTNAT^−/−^* males. Dissociated eupyrene sperm flagella carried punctate protrusions (Figure 5E), and the number of eupyrene sperm in the bursa copulatrix was markedly reduced (Figure 5F). We also found that loss of *LTNAT* did not affect the individualization of eupyrene sperm bundles (Figure S15, Supporting Information). Analysis of spermathecae 24 h after mating revealed a striking difference: spermathecae of females mated with WT males were filled with sperm, whereas those of females mated with *LTNAT^−/−^* males appeared translucent and lacked sperm (Figure 5G,H). Based on previous studies [10], we hypothesized that this phenotype might be caused by the decreased motility of apyrene sperm. To test this, we used video microscopy to record the motility of apyrene sperm isolated from the spermatophore. Compared with WT apyrene sperm (Movie S1, Supporting Information), the motility of apyrene sperm in *LTNAT^−/−^* males was severely impaired (Movie S2, Supporting Information).

Unlike *Sxl* mutants [10], in which apyrene sperm development is disrupted, apyrene sperm in *LTNAT^−/−^* males developed normally but exhibited reduced motility. Finally, we asked whether LTNAT, like many seminal fluid proteins, is transferred to the female bursa copulatrix and acts there. The LTNAT signal was not detected in the bursa copulatrix or spermatheca of virgin females and the spermatozoa matrix (Figure S16, Supporting Information). Additionally, LTNAT lacks a signal peptide and is not expressed in male accessory glands (Figure 1E,F). Together, these observations indicate that LTNAT functions as a sperm protein and that its absence directly causes functional defects in apyrene sperm. Overall, our results demonstrate that LTNAT is critical for the function of dimorphic sperm.

### 2.6 *LTNAT* Mutation Disrupts the Structure of Mitochondrial Derivatives in Eupyrene Sperm

**FIGURE 6:**
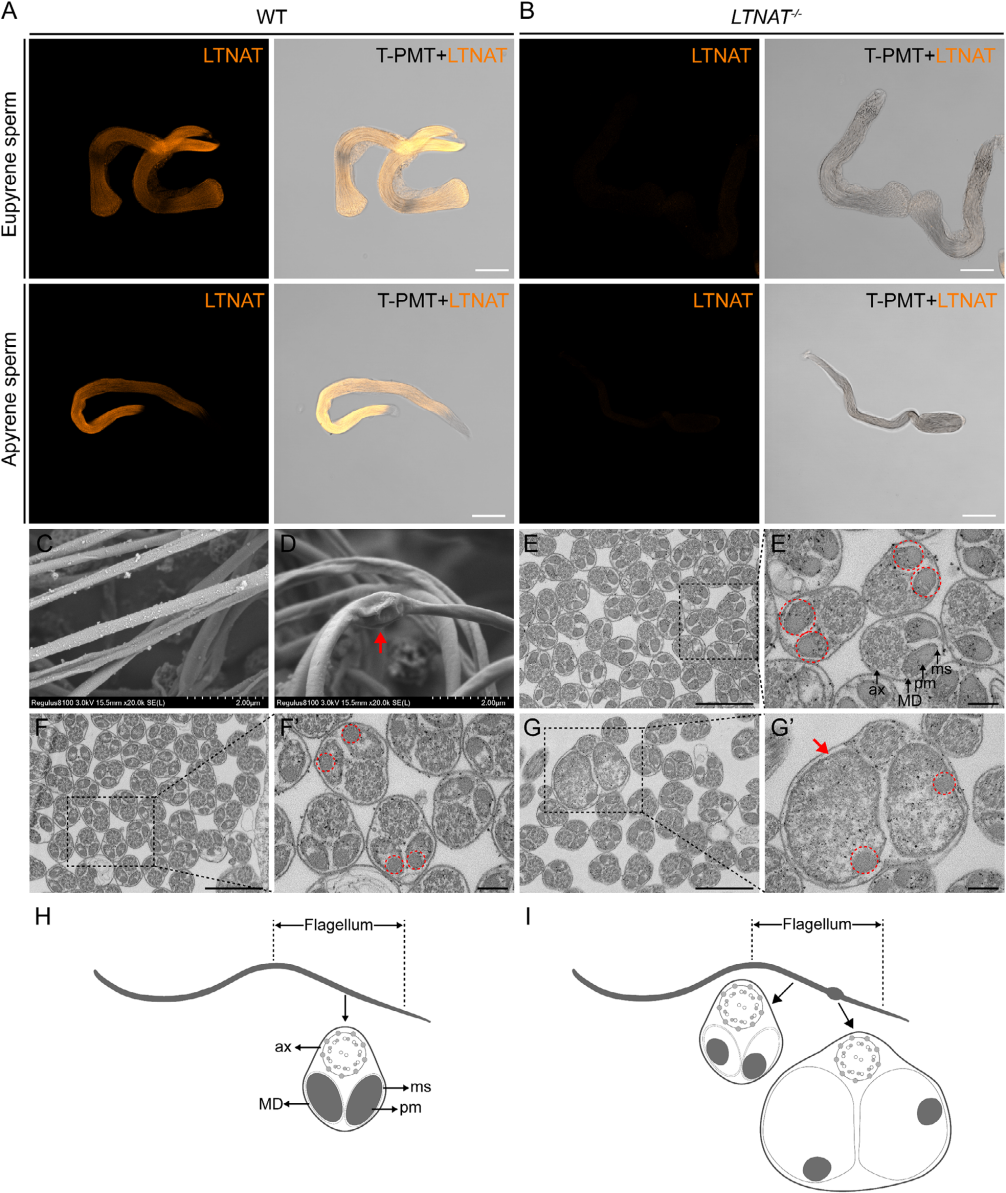
*LTNAT* deficiency disrupts MDs' integrity in eupyrene sperm. (A) Immunolocalization of LTNAT (orange) in eupyrene sperm and apyrene sperm of WT males. Scale bar: 20 µm. (B) Immunolocalization of LTNAT (orange) in apyrene sperm of *LTNAT^−/−^* males. Scale bar: 20 µm. (C) SEM image of WT eupyrene sperm. Scale bar: 2 µm. (D) SEM image of eupyrene sperm in *LTNAT^−/−^* males. The red arrow points to the irregular bulges. Scale bar: 2 µm. (E) TEM analysis of the cross‐section of the flagella of WT eupyrene sperm. Scale bar: 1 µm. (E′) High‐magnification view (dashed box in E): Flagellar ultrastructure showing two MDs with paracrystalline material (pm), wrapped by membrane structure (ms), and the 9+9+2 axoneme (ax). The red circle marks the size of pm. Scale bar: 200 nm. (F) TEM analysis of the cross‐section of the flagella of *LTNAT^−/−^* eupyrene sperm. Scale bar: 1 µm. (F′) High‐magnification view (dashed box in F): The accumulation of pm decreases. The red circle marks the size of pm. Scale bar: 200 nm. (G) TEM analysis of the swollen sperm flagellar structure in *LTNAT^−/−^* males. Scale bar: 1 µm. (G′) The magnified image (dashed box in G) shows the damage to the ms of MDs and the swelling of MDs. The red arrow points to the swollen MDs, and the red circle indicates the size of the pm. Scale bar: 200 nm. (H) Schematic model of the cross‐sectional structure of the flagella of WT eupyrene sperm. (I) Schematic model of the cross‐sectional structure of the flagella of *LTNAT^−/−^* eupyrene sperm.

To clarify how the *LTNAT* mutation affects sperm function, we first examined the subcellular localization of LTNAT in sperm by immunofluorescence. LTNAT localized to both eupyrene and apyrene sperm, with the strongest signal in the flagellar region of eupyrene sperm (Figure 6A). In sperm from *LTNAT^−/−^* males, however, specific LTNAT signals were almost undetectable, confirming efficient gene knockout (Figure 6B). We next asked whether loss of *LTNAT* alters the outer envelope of sperm bundles. Scanning electron microscopy (SEM) analysis showed that the surfaces of eupyrene and apyrene sperm bundles in *LTNAT^−/−^* males remained smooth and compact, with no obvious differences from WT bundles (Figure S17, Supporting Information). By contrast, SEM observation of dissociated eupyrene sperm in the bursa copulatrix revealed that, relative to the straight and smooth flagella in WT males, eupyrene sperm flagella from *LTNAT^−/−^* males displayed irregular, localized swellings (Figure 6C,D).

On this basis, we analyzed sperm from WT and *LTNAT^−/−^* males by transmission electron microscopy (TEM) to further characterize flagellar ultrastructure. In WT eupyrene sperm, the flagellum consists of a typical “9+9+2” axoneme and two mitochondrial derivatives (MDs) of uniform size, each enclosed by a membrane structure and filled with dense paracrystalline material (Figure 6E,H). In *LTNAT* mutants, the “9+9+2” axonemal arrangement of eupyrene sperm remained intact, but the ultrastructure of the MDs was markedly altered: (i) paracrystalline material was poorly deposited (Figure 6F,I), (ii) the membranes surrounding the MDs were disrupted (Figure 6G,I), and (iii) the MDs were strongly swollen (Figure 6G,I). These abnormalities are consistent with the focal swellings observed along the flagellum by SEM. TEM analysis of apyrene sperm showed that the cross‐sections from WT and *LTNAT^−/−^* males were similarly elliptical and contained a single “9+9+2” axoneme and two relatively small MDs, indicating that the *LTNAT* mutation did not affect the ultrastructure of apyrene sperm (Figure S18, Supporting Information).

### 2.7 *LTNAT* Knockout Induces Mitochondrial Dysfunction and Inhibits Motor Proteins

**FIGURE 7:**
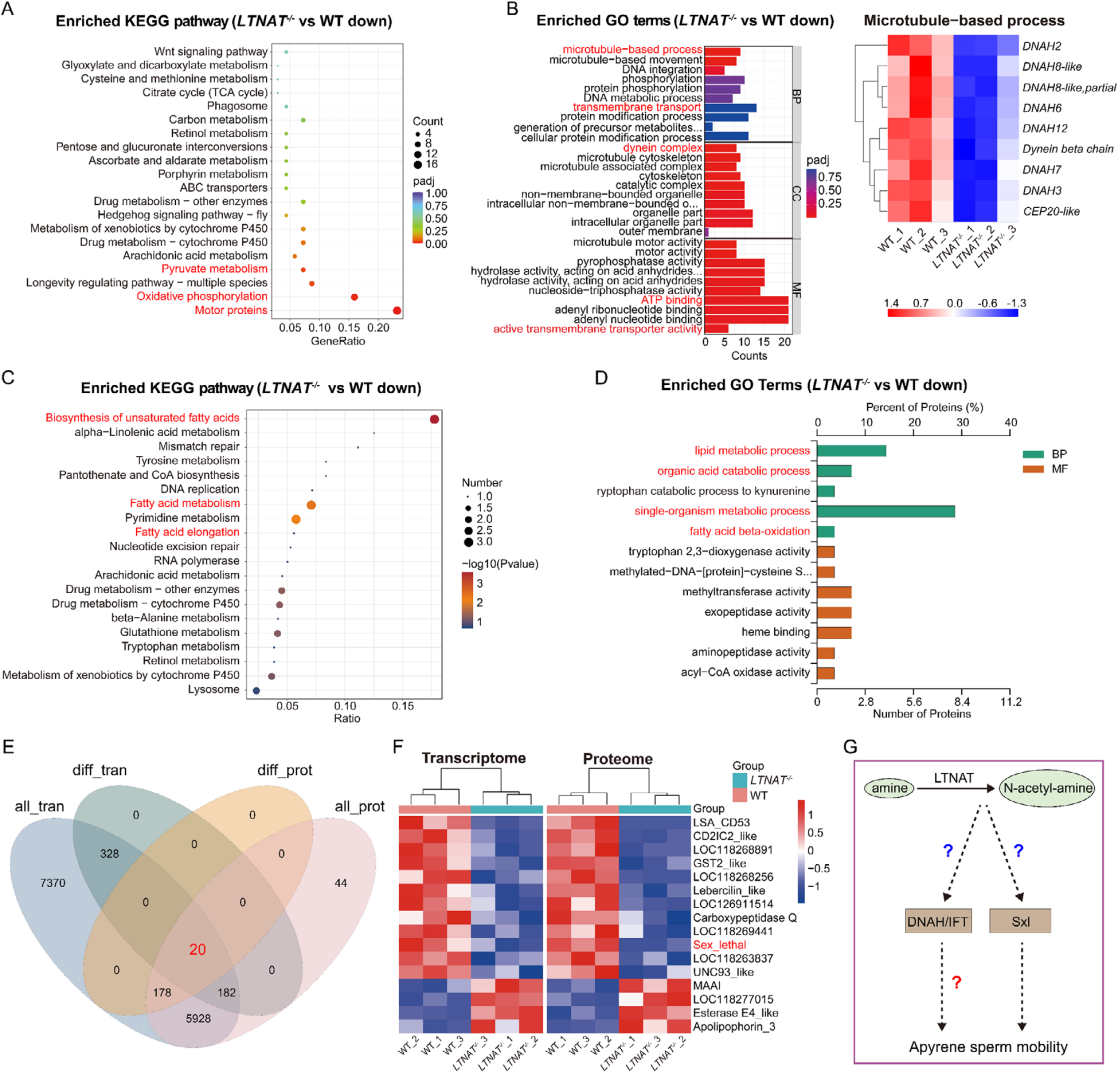
Integrated transcriptomic and proteomic analyses reveal *LTNAT* mutation impairs mitochondrial metabolism and motor proteins. (A) KEGG pathway analysis of downregulated genes in *LTNAT^−^* testes shows enrichment in motor proteins, oxidative phosphorylation, and pyruvate metabolism. (B) GO analysis of downregulated genes in *LTNAT^−/−^* testes reveals significant enrichment for microtubule‐based processes, transmembrane transport, and ATP binding. The right panel displays a heatmap of genes associated with microtubule‐based processes (red: high expression; blue: low expression). (C) KEGG pathway enrichment analysis shows that proteins in *LTNAT^−/−^* testes with decreased abundance are involved in fatty acid metabolism and unsaturated fatty acid biosynthesis. (D) Proteins downregulated in *LTNAT^−/−^* testes are enriched in GO terms related to lipid metabolism. (E) Venn diagram showing overlapping DEGs and DEPs. (F) Heatmap of 16 genes that are consistently down‐ or upregulated in both transcriptomic and proteomic profiles. The Sex‐lethal gene is shown in red. Red indicates high expression, and blue indicates low expression. (G) The schematic diagram of the proposed hypothesis shows that LTNAT regulates the expression of *Sxl*, *DNAH*, and *IFT* by maintaining the homeostasis of biogenic amine metabolism, thereby maintaining the motility of apyrene sperm. The blue question mark indicates how LTNAT regulates the expression of *Sxl*, *DNAH*, and *IFT*. The red question mark indicates whether DNAH/IFT family genes also regulate the motility of apyrene sperm in lepidopteran insects.

To elucidate how LTNAT regulates MD formation and sperm motility, we performed integrated transcriptomic and proteomic analyses on testes from 1‐day‐old WT and *LTNAT^−/−^* males. Transcriptome profiling identified 556 DEGs, including 164 upregulated and 392 downregulated genes (Figure S19A,B, Supporting Information). KEGG and GO enrichment analyses showed that downregulated genes in *LTNAT^−/−^* males were mainly associated with energy metabolism, microtubule‐based processes, and transmembrane transport (Figure 7A,B). Notably, several *axonemal dynein heavy chain* (*DNAH*) genes involved in microtubule‐based motility were strongly downregulated (Figure 7B), and these genes are known to play key roles in sperm motility in mice [25, 26, 27, 28, 29, 30]. Consistent with impaired mitochondrial energy metabolism, ATP levels were significantly reduced in *LTNAT^−/−^* sperm (Figure S20, Supporting Information).

At the proteomic level, we quantified 6521 proteins, of which 64 were significantly downregulated, and 137 were significantly upregulated (Figure S21, Supporting Information). Subcellular localization analysis showed that differentially expressed proteins (DEPs) between *LTNAT^−/−^* and WT males were predominantly mitochondrial (25.93%) (Figure S22A, Supporting Information), consistent with the ultrastructural abnormalities observed in MDs. Integrated KEGG and GO analyses indicated that downregulated proteins were significantly enriched in lipid metabolic processes closely linked to energy production and membrane biosynthesis (Figure 7B,C). Gene set enrichment analysis (GSEA) further revealed marked downregulation of proteins involved in microtubule‐based movement, mainly DNAH proteins (Figure S22B, Supporting Information). Protein–protein interaction network analysis of downregulated proteins identified three functionally coherent modules: detoxification and lipid metabolism, cellular homeostasis, and cilium/flagellum assembly (Figure S22C, Supporting Information). Notably, the flagellar module contained intraflagellar transport (IFT) complex subunits IFT74, IFT88, and IFT172, which are essential for axoneme assembly and sperm motility in mice [31, 32, 33, 34]. By contrast, genes and proteins upregulated in *LTNAT^−/−^* males were enriched in oxidation‐reduction processes, diverse metabolic pathways, and DNA integration (Figures S19C–D and S21C–D, Supporting Information). The results suggest that cells undergo compensatory metabolic reprogramming in response to energy depletion and membrane lipid damage to limit oxidative injury and genotoxic stress.

In the integrated multi‐omics analysis, we identified 20 genes that were differentially expressed at both the transcript and protein levels, among which 16 showed concordant changes in expression (Figure 7E,F). Notably, *Sxl* was significantly downregulated in *LTNAT^−/−^* males (Figure 7F). Genetic studies in the silkworm have shown that *Sxl* mutation causes abnormal apyrene sperm development and loss of motility [10, 12]. *Sxl* also regulates apyrene sperm bundle formation in *S. litura* and male fertility in *S. frugiperda* [35, 36]. These findings suggest that the role of *Sxl* in apyrene sperm development and motility may be conserved among lepidopteran insects. Given the conserved functions of key sperm genes between mice and Lepidoptera [13, 37], we hypothesize that *LTNAT* knockout may reduce apyrene sperm motility by downregulating *DNAH* and *IFT* genes expression. Overall, our multi‐omics data support a model in which *LTNAT* mutation disrupts mitochondrial energy metabolism and membrane lipid homeostasis, likely causing osmotic imbalance and abnormalities in MDs. Meanwhile, *LTNAT* knockout may impair the motility of apyrene sperm by reducing the expression of *Sxl* and DNAH/IFT family genes (Figure 7G).

### 2.8 Excessive Release of *LTNAT^−/−^* Males Inhibits Female Fertility

**FIGURE 8:**
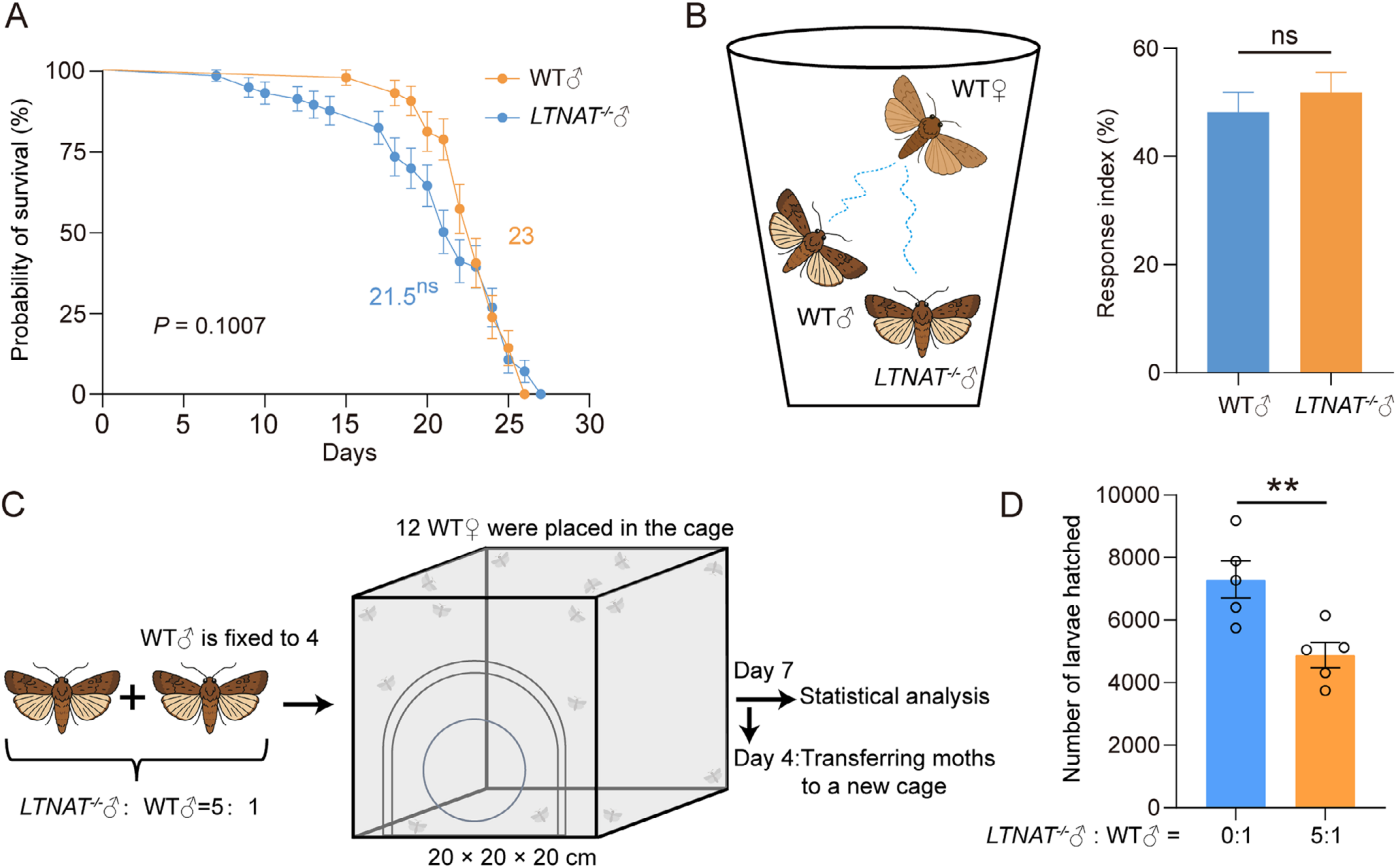
Cage release of *LTNAT^−/−^* males causes cage population suppression. (A) Survival curves of WT males and *LTNAT^−/−^* males. Log‐rank (Mantel‐Cox) test; *p* values are shown in the picture. *n* = 42 for WT and *n* = 56 for *LTNAT^−/−^*. (B) Mating success rates in competitive assays. *n* = 3 biological replicates (10 males per group). Student's *t‐*test; ns, not significant. (C) Experimental design for competitive population suppression assay. (D) Viable offspring counts. *n* = 5 biological replicates. Student's *t‐*test; **p < 0.01.

Finally, we evaluated whether mass‐released *LTNAT^−−^* males can suppress population growth under caged conditions. Fitness cost analysis showed that the *LTNAT* mutation did not significantly affect male lifespan or mating competitiveness (Figure 8A,B). We then introduced *LTNAT^−/−^* and WT males at a ratio of 5:1 into cages containing 12 virgin females (Figure 8C). Compared with control cages containing only WT males, cages with excess *LTNAT^−/−^* males produced significantly fewer offspring (Figure 8D). These findings indicate that *LTNAT* is a promising molecular target for the genetic control of lepidopteran pests.

## 3 Discussion

In this study, we identified and functionally characterized a lepidoptera‐conserved, testis‐specific arylalkylamine *N*‐acetyltransferase (LTNAT) in *S. frugiperda*. Loss of *LTNAT* induces abnormal morphology of MDs in eupyrene sperm and decreased motility of apyrene sperm. Integrated multi‐omics analyses further indicated that *LTNAT* is a key factor in maintaining sperm cells' homeostasis of energy production, lipid metabolism, and transmembrane transport. At the expression regulation level, *LTNAT* mutation may impair apyrene sperm motility by downregulating the expression of *Sxl* and DNAH/IFT family genes. Together, these findings establish *LTNAT* as a central regulator of male fertility in moths and suggest a potential mechanism controlling dichotomous spermatogenesis.

With the rapid development of omics and gene editing technologies, an increasing number of genes involved in reproductive development have been identified [38, 39, 40]. Here, we first compared the transcriptomic profiles of Te, MAG, and MCa of 1‐day‐old adult males to screen for testis‐specific genes. In combination with CRISPR/Cas9‐mediated gene editing, we identified a previously uncharacterized male sterility gene, *LTNAT*. Although insect aaNATs show generally low amino acid sequence identity, their tertiary structures are highly conserved across the family [41]. Consistent with this, LTNAT shares low sequence identity with the DmagmNAT but has a highly similar overall fold and conserved motif organization. Among these, motif A and motif B form the binding sites for acetyl‐CoA and the substrate, respectively, whereas motif C and motif D stabilize the overall protein structure [42, 43, 44, 45]. In addition, LTNAT possesses another typical feature of insect aaNATs: a helix/helices element located between β3 and β4. Insects possess multiple aaNATs to regulate the metabolism of biogenic amines and the biosynthesis of fatty acid amides [41, 46, 47, 48]. Using a DTNB colorimetric assay, we further showed that recombinant LTNAT catalyzes *N*‐acetylation of two classes of biogenic amines: polyamines and aromatic amines. Similar to TcaaNAT1 [47], LTNAT exhibits a relatively broad substrate spectrum, suggesting that its function may extend beyond a single metabolic pathway. However, in this study, we only performed preliminary characterization of LTNAT activity at a single substrate concentration, and its kinetic parameters and detailed catalytic mechanism remain to be determined. Previous work has shown that insect aaNATs play important roles in neurotransmitter inactivation [49], pigment deposition [50, 51, 52], cuticle sclerotization [53, 54], and ovarian development [51, 52, 55]. In contrast, we found that LTNAT specifically regulates male fertility in moths and that its loss leads to complete male sterility, providing a new perspective on functional diversification within the insect aaNATs family.

Because eupyrene sperm are responsible for fertilization, defects in their development or transfer often directly lead to male sterility [15, 19]. Previous studies have identified multiple key genes that specifically control nuclear positioning in eupyrene sperm and the migration of eupyrene sperm into the female reproductive tract [10, 15, 16, 17, 19]. In contrast, *LTNAT* mutation neither alters nuclear positioning in eupyrene sperm nor completely blocks their transfer to females during mating. Instead, we discovered a previously unreported phenotype: loss of *LTNAT* causes pronounced punctate swellings along eupyrene sperm flagella. Immunocytochemical analyses showed that LTNAT is expressed predominantly in eupyrene sperm, with the strongest signals in the flagellar region. Combined SEM and TEM revealed that in *LTNAT^−/−^* eupyrene sperm, the paracrystalline material was reduced, the membranes surrounding the MDs were disrupted, and the MDs became swollen. Mitochondria are integral components of the sperm flagellum and play central roles in maintaining flagellar structural integrity and supplying energy for beating [56, 57, 58]. Studies in multiple species have shown that mitochondrial dysfunction often causes male sterility [37, 39, 57, 59]. For example, *S‐Lap* mutants in flies exhibit male sterility due to defects in paracrystalline material accumulation [59], and loss of *TSSKL* results in altered mitochondrial morphology and reduced sperm motility [37]. Because eupyrene sperm cannot actively migrate through the female reproductive tract, we speculate that MDs of eupyrene sperm primarily provide energy reserves and structural support for egg activation or early embryonic development. Future in‐depth analysis of LTNAT‐related omics data to identify downstream factors specifically involved in eupyrene MDs formation may provide direct genetic evidence for this hypothesis. Further, enrichment analyses showed that genes involved in energy metabolism, membrane lipid synthesis, and transmembrane transport are significantly downregulated in *LTNAT^−/−^* males. We therefore infer that loss of *LTNAT* impairs mitochondrial energy production and disrupts membrane lipid integrity, collectively increasing osmotic pressure within MDs and leading to water influx and swelling.

Motile apyrene sperm assist eupyrene sperm in transferring from the bursa copulatrix to the spermatheca [10, 12]. Therefore, impairment or loss of apyrene sperm motility also causes male sterility [10, 12]. Consistent with previous studies, we found that knockout of *LTNAT* significantly reduces apyrene sperm motility and prevents eupyrene sperm from reaching the spermatheca. Immunohistochemical analyses confirmed that LTNAT is also expressed in apyrene sperm. However, unlike the ultrastructural defects observed in eupyrene sperm, the absence of LTNAT did not alter the external morphology or the internal axoneme and MDs structure of apyrene sperm. Omics data further showed that multiple members of the DNAH and IFT protein families are downregulated in *LTNAT^−/−^* testes. DNAH and IFT proteins are essential for maintaining flagellar structural integrity and effective sperm motility in mammals [25, 26, 27, 28, 29, 30, 31, 32, 33, 34]. Although the reproductive systems of insects and mammals differ anatomically, the genetic basis of spermatogenesis is partly conserved [13, 37]. For instance, the *TSSK* gene regulates spermatogenesis in both mice and the silkworm [37]. Moreover, the core architecture and driving mechanism of sperm and cilia are highly conserved across animals [60, 61, 62]. Therefore, from a mechanistic perspective, we hypothesize that *LTNAT* knockout indirectly compromises apyrene sperm motility by reducing the expression levels of DNAH/IFT family genes. We also observed that *Sxl* expression is significantly downregulated in the *LTNAT* mutants. Previous studies have shown that *Sxl* mutation causes MD defects in apyrene sperm and complete loss of motility [10]. However, apyrene sperm of *LTNAT^−/−^* males retain normal MDs but show impaired beating. We believe that the consequences of *Sxl* downregulation levels differ between these genetic contexts.

Seminal fluid proteins (SFPs) are transferred to the female reproductive tract during mating and trigger a range of physiological and behavioral responses [21]. Several studies have indicated that SFPs are critical for apyrene sperm activation [22, 63]. Therefore, we considered whether LTNAT functions as a seminal fluid protein within the spermatophore. SFPs are mainly synthesized in the male accessory glands and mix with sperm in the double ejaculatory ducts [64]. Our data showed that *LTNAT* is not expressed in male accessory glands and is not transferred to spermatophores. In addition, *LTNAT* mutation directly induces marked morphological changes in eupyrene sperm. These observations suggest that LTNAT more likely acts as an intrinsic sperm protein involved in the development and functional regulation of dimorphic sperm.

The precise molecular mechanisms of how LTNAT regulates spermatogenesis remain unclear, largely due to the imbalance of the metabolic homeostasis of polyamines in sperm cells. Polyamines are highly abundant in testes and sperm, where they bind strongly to nucleic acids and negatively charged membrane lipids [65, 66]. Dysregulated polyamine abundance can disrupt normal spermatogenesis and sperm motility [67, 68]. Based on our multi‐omics data and existing literature, we propose the following working hypotheses for LTNAT's regulatory mechanisms in dichotomous spermatogenesis: 1) In eupyrene sperm: Loss of LTNAT function may lead to excessive accumulation of positively charged polyamines within mitochondrial derivatives. This accumulation could disrupt membrane curvature, impair the ordered arrangement of mitochondrial cristae, and interfere with the deposition of paracrystalline materials—ultimately resulting in the mitochondrial derivative swelling and paracrystalline material reduction. 2) In apyrene sperm: LTNAT may regulate polyamine signaling pathways to modulate the transcription or stability of multiple motility‐related factors, including Sxl and cilia/flagella assembly proteins. The combined reduction of these factors may collectively weaken apyrene sperm motility. Future studies integrating metabolomics with transcriptomics will be essential to identify LTNAT's endogenous substrates and to determine how such metabolic perturbations ultimately impact the expression of motility genes and apyrene sperm function.

CRISPR/Cas9‐based precision‐guided SIT (pgSIT) holds great promise for pest management [69, 70]. Identifying male sterility genes targeting sperm proteins in lepidopteran pests will provide molecular targets for pgSIT or other sterile male‐based techniques [71, 72, 73, 74, 75]. Multiple cage experiments have shown that releasing excess sterile males can substantially reduce offspring production by WT females [19, 76, 77]. We first evaluated the impact of *LTNAT* mutation on male survival and mating competitiveness, two key traits for establishing pgSIT systems [69]. Our results showed that *LTNAT* mutation did not compromise male viability or mating capacity, and that mass release of *LTNAT^−/−^* males under competitive mating conditions significantly reduced offspring numbers. These findings indicate that *LTNAT* is a promising molecular target for pest control. Because LTNAT is highly conserved among lepidopteran insects but shows low sequence identity to other insect orders, it also has strong potential as an ideal target for highly specific insecticides [78]. Future work will exploit the detailed structure and catalytic properties of LTNAT to enable high‐throughput screening for small‐molecule inhibitors.

## 4 Experimental Section

### 4.1 Insect Rearing

The *S. frugiperda* and *S. exigua* strains were obtained from Keyun Biology Co., Ltd. The larvae were raised on an artificial diet. Each larva was kept individually in a plastic tube (20 × 100 mm) and maintained in an incubator at 26 ± 1°C, 65 ± 5% relative humidity, and a 14:10 h light: dark cycle. Pupae were sexed based on morphological features and kept under the same conditions as the larvae. When the pupae turned dark, they were transferred to cages measuring 20 × 20 × 20 cm for adult emergence. Adults were kept in an incubator at 26 ± 1°C, 75 ± 5% relative humidity, and a 14:10 h light: dark cycle, and were fed daily with a 10% honey solution.

The *C. suppressalis* strain was provided by Dr. Han Lan‐Zhi (Chinese Academy of Agricultural Sciences). The larvae were fed with artificial feed and kept in an incubator at a temperature of 28 ± 1°C, a relative humidity of 65 ± 5%, and a photoperiod of 16 h light: 8 h dark. The adults were reared in an incubator with a relative humidity of 85 ± 5% and were fed with 10% honey water as a nutrient source.

### 4.2 RNA Isolation and RT‐PCR

Total RNA was extracted from the testes of 1‐day‐old male adults using VeZol Reagent (R44‐02; Vazyme Biotech, Nanjing, China). First‐strand complementary DNA (cDNA) was synthesized using the HiScript III first Strand cDNA Synthesis Kit with gDNA wiper (Vazyme Biotech). The open reading frame (ORF) of the target gene was predicted using the online tool NCBI ORF Finder. Primers for gene cloning and quantitative analysis were designed based on ORF Finder results. PCR amplification and RT‐PCR were performed using 2× Phanta Max Master Mix (P515‐01; Vazyme Biotech, Nanjing, China). For RT‐PCR, each RNA sample was used at a concentration of 500 ng/µL. *RpL18* was used as the internal reference gene.

### 4.3 Bioinformatics Analysis

Potential signal peptides were predicted using SignalP 6.0 (https://services.healthtech.dtu.dk/services/SignalP‐6.0/), and transmembrane domains were identified with DeepTMHMM 1.0 (https://services.healthtech.dtu.dk/services/DeepTMHMM‐1.0/). Conserved protein domains were analyzed via NCBI CD‐Search (https://www.ncbi.nlm.nih.gov/Structure/cdd/wrpsb.cgi) and SMART (http://smart.embl‐heidelberg.de/). The molecular weight of LOC118263478 is predicted by an online software Expasy (http://web.expasy. org/protparam/). The LOC118263478 structure was modeled using AlphaFold Monomer v2.0. Discover the structures similar to LOC118263478 from the Protein Data Bank [79, 80]. Multiple sequence alignments were performed using ClustalX 1.81, and results were visualized with ESPript 3.2 (https://espript.ibcp.fr/ESPript/cgi‐bin/ESPript.cgi). To find homologs of LTNAT in insects, the LTNAT protein sequence was used as a query for tBLASTn and BLASTp searches against genomic and transcriptomic databases, including GenBank and InsectBase 2.0. The species phylogenetic tree was built using TimeTree 5, with representative insects from other orders except Lepidoptera serving as the outgroup [81]. To analyze the evolutionary relationships of putative LTNAT orthologs in Lepidoptera and those of the aaNATs family, amino acid sequences were aligned with ClustalW (the longest isomers were used for phylogenetic analysis), and a maximum likelihood phylogenetic tree was created using MEGA X.

### 4.4 Transcriptome Analysis

To identify genes specifically expressed in the testes, total RNA was extracted from the testis (Te), Male accessory glands (MAGs), and remaining male tissues excluding the Te and MAGs of 1‐day‐old male adults for transcriptomic analysis. To analyze gene expression changes caused by *LTNAT* knockout, total RNA was extracted from testes of the same size in 1‐day‐old WT and mutant males for RNA‐seq analysis. Each group included three biological replicates, with each replicate consisting of 10 pooled tissues. RNA quality and concentration were assessed with a spectrophotometer (NanoDrop 2000, Thermo, Shanghai, China). RNA quantity and integrity were further verified using an Agilent 2100 Bioanalyzer. Messenger RNA (mRNA) was enriched from total RNA using Oligo(dT) magnetic beads. After fragmentation, first‐strand and second‐strand cDNA were synthesized using random hexamer primers. The double‐stranded cDNA was then subjected to end repair, A‐tailing, adaptor ligation, size selection, amplification, and purification. Sequencing was carried out on the Illumina NovaSeq 6000 platform. Raw reads were processed with fastp to remove adapters, poly‐N reads, and low‐quality sequences, producing clean reads. A reference genome index was built with HISAT2, and the paired‐end clean reads were aligned to the reference genome. Novel genes were predicted using StringTie (v2.2.3). Gene‐level read counts were calculated with featureCounts (v2.0.6), and FPKM (Fragments Per Kilobase of transcript per Million mapped reads) values were calculated based on gene length and read counts. Differential expression analysis between groups was performed using the DESeq2 R package (v1.42.0). Genes with an adjusted *p*‐value (padj) ≤ 0.05 and |log_2_(fold change)|≥1 were considered significantly differentially expressed.

In Interproscan software, protein sequences from identified genes are aligned (evalue ≤ 1e−5) against the Pfam database to obtain Gene Ontology (GO) annotation information. In the kobas‐3.0.3 software, protein sequences from identified genes are subjected to BLAST (blastp, evalue ≤ 1e−5) against the Kyoto Encyclopedia of Genes and Genomes (KEGG) database of the lepidopteran model organism, the silkworm. For each sequence's BLAST result, the highest‐scoring match is selected to obtain the KEGG annotation. GO and KEGG enrichment analyses were performed on DEGs using the hypergeometric test with clusterProfiler (v4.8.1), defining significant enrichment as a padj ≤ 0.05.

### 4.5 Generation of Mutants

To improve gene editing efficiency, two highly effective guide RNAs (gRNAs) targeting the gene of interest were designed based on established principles [82]. The Cas9 protein used was a commercial product, TrueCut Cas9 Protein v2 (Thermo Fisher Scientific, USA). sgRNAs were synthesized in vitro using the GeneArt Precision gRNA Synthesis Kit (Thermo Fisher Scientific, Vilnius, Lithuania). The gene editing protocol for *S. frugiperda* and *S. exigua* followed previously published methods [83]. Briefly, ten pairs of 2‐day‐old adult moths were placed in a plastic box lined with gauze for mating and oviposition. After entering the dark period, fresh egg masses were collected every 30 min (m). Dispersed individual eggs were transferred to double‐sided adhesive tape on glass slides with a fine brush. The Cas9 protein (300 ng/µL) and sgRNA (150 ng/µL) mixture was injected into the eggs using the InjectMan NI 2 microinjection system (Eppendorf, Hamburg, Germany). Injected eggs and hatched larvae were maintained in a climate chamber at 26 ± 1°C, 70% ± 5% relative humidity, and a 14:10 h light/dark photoperiod. PCR primers flanking the sgRNA target site were designed to detect mosaic mutations in G_0_ individuals. DNA was extracted from the hind leg of G_0_ adults for PCR amplification and Sanger sequencing. Overlapping chromatogram peaks at the target region were considered indicative of mutation. G_0_ mutants were crossed with WT moths to produce the G_1_ generation. To evaluate heritability, DNA from a pool of 20 randomly selected G_1_ larvae was extracted and analyzed. PCR amplicons from G_1_ individuals were cloned into pClone007 Blunt Simple Vector (TSINGKE, Beijing, China), and positive clones were sequenced to determine whether the mutation caused a premature stop codon. G_1_ individuals carrying the same effective frameshift mutation were interbred to establish homozygous mutant lines.

The egg collection and microinjection procedures for *C. suppressalis* followed our previous study [84]. Briefly, fresh egg masses were collected from 30‐day‐old rice seedlings within 2 h of oviposition. The egg masses were transferred to slides and injected as described above. The same concentrations of Cas9/sgRNA, injection equipment, and genotyping strategy used for *S. frugiperda* were applied. On the second day after injection, the injected eggs were immersed in a 10% formaldehyde solution for 15 m and then rinsed with sterile water. The injected eggs were placed in a petri dish with five layers of wet filter paper and incubated at 28 ± 1°C and a relative humidity of 65% ± 5%.

### 4.6 Fertility and Fecundity Assessment

To evaluate the effect of gene mutation on male reproductive performance, 2–3‐day‐old virgin males and females were individually paired in 490 mL plastic cups. After the start of the dark period, mating behavior was observed under red light at 15 m intervals. Mating success rate and copulation duration were recorded. Following mating, eggs laid by each female were collected daily for four consecutive days. The total number of eggs, the number of hatched offspring, and the hatching rate were measured. All experiments were conducted in a climate‐controlled chamber set at 26 ± 1°C, 75% ± 5% relative humidity, with a 14 h light and 10 h dark photoperiod. Adults were fed a 10% honey solution as a nutritional source.

To assess the impact of gene mutations on male reproductive capacity in *C. suppressalis*, we also conducted single‐pair mating experiments. One‐day‐old virgin males and females were paired in 350 mL plastic cups for mating. After copulation, eggs laid by each female were collected daily for three days. Seven days after oviposition, the number of hatched larvae was counted to evaluate male reproductive ability. All experiments were performed in a climate‐controlled incubator maintained at 26 ± 1°C with 85% ± 5% relative humidity, under a 14:10 h light/dark cycle. A 10% honey solution was provided daily.

### 4.7 Protein Expression and Purification

The methods for heterologous expression and Ni‐NTA affinity enrichment of LOC118263478 protein refer to the published articles [85]. The *LOC118263478* gene was cloned into the pET30a (+) vector. Sequence‐verified plasmids were transformed into *E. coli* BL21(DE3) competent cells. A single positive colony was inoculated into 100 mL LB medium supplemented with kanamycin (50 µg/mL) and cultured at 37°C with shaking (220 rpm) until OD_600_ reached 0.6 ± 0.1. Protein expression was induced with 0.5 mM isopropyl β‐D‐1‐thiogalactopyranoside (IPTG) at 16°C for 24 h with agitation (170 rpm). Cells were harvested by centrifugation (8,000 × *g*, 10 min, 4°C), washed twice with phosphate‐buffered saline (PBS; pH 7.4), and resuspended in 10 mM Tris‐HCl buffer (pH 8.0). Cells were lysed by sonication on ice (20 kHz, 3 s pulse/6 s interval, 20 min total duration). The lysate was clarified by centrifugation (12,000 × *g*, 30 min, 4°C). 15 µL Supernatant was mixed with 5 µL 4× Laemmli loading buffer, boiled for 5 min, and analyzed via 4%–20% SDS‐PAGE. The supernatant was loaded onto a pre‐equilibrated Ni‐NTA agarose column (1 mL bed volume) at a 1 drops/s flow rate. Impurities were removed with Buffer A (20 mM Tris‐HCl, 500 mM NaCl, 20 mM imidazole, pH 8.0). Bound proteins were eluted using a 50 mM imidazole solution. Fractions containing the predominant band at the expected molecular weight of recombinant LOC118263478 (∼35 kDa, including tag) were pooled, dialyzed against 0.05 M PBS (pH 7.2) using a 14 kDa MWCO membrane (Solarbio, China; Cat#D8350) with three buffer changes at 6 h intervals (4°C). The dialysate was concentrated with polyethylene glycol 20,000 (PEG 20,000). Protein concentration determined by A280 represents total protein in the enriched fraction. Protein concentration was adjusted to 0.4 mg/mL and stored at −80°C.

### 4.8 Enzyme Assay

Since the acetylation process involves the transfer of an acetyl group from acetyl‐coenzyme A (CoA) to a substrate, this exposes the sulfhydryl group on CoA. Catalytic activity was determined by measuring CoA release during acetyl transfer from acetyl‐CoA to biogenic amine substrates using 5,5′‐dithiobis‐(2‐nitrobenzoic acid) (DTNB) as chromogenic agent [86, 87, 88]. Reactions contained in 100 µL final volume: 50 mM Tris‐HCl (pH 7.3), 1.0 mg mL^−1^ BSA, 4 mM substrate, and 0.4 µg purified LOC118263478. After 5 min pre‐incubation at 25°C, reactions were initiated with 0.4 mM acetyl‐CoA. Following 5 min incubation, 25 µL DTNB stop solution (5 mM DTNB in 6.4 M guanidine HCl/0.1 M Tris‐HCl, pH 7.3) was added. The absorbance at 412 was measured within 5 min (SpectraMax M5e, Molecular Devices). Recombinant protein was omitted to serve as controls. Specific activity (nmol min^−1^ mg^−1^) was calculated using ε(412 nm) = 13700 M^−1 ^cm^−1^ for TNB^−^ [88]. *l* is equal to 0.5 cm. Lambert Beer equation: *c* = Δ*Abs*/ε*l.

### 4.9 Sperm Staining

Sperm staining was performed following previously described protocols [15, 19]. Briefly, sperm samples were fixed in 1 mL of 4% paraformaldehyde (R22039, Shanghai Yuanye Bio‐Technology Co., Ltd.) for 1 h at room temperature. The fixed samples were washed thrice with PBS, each lasting 10 m. Sperm were then incubated in 400 µL of TRITC‐phalloidin (1:1000 dilution; 40734ES75, YEASEN Biotechnology Co., Ltd.) for 1 h, followed by incubation with 400 µL of DAPI (1:1000 dilution; C0060, Solarbio, Beijing, China) for 10 m. After staining, the samples were washed three times with PBST (PBS with 0.1% Tween‐20), each wash lasting 10 m. A 10 µL aliquot of the stained sperm suspension was mounted on a glass slide, covered with a coverslip, and gently pressed to spread the sample evenly. For the statistics of sperm count, we image the samples using the five‐point sampling method under a 20× field of view. Fluorescence images were acquired using a Zeiss LSM 980 confocal microscope equipped with Airyscan 2 technology. Image processing and analysis were performed with ImageJ software.

### 4.10 Immunofluorescence Staining

A polyclonal antiserum was produced in rabbits using the C‐terminal peptide sequence (SKTATRNSFIEPGN) of the LTNAT protein. The antiserum was supplied by Hangzhou Huaan Biotechnology Co., Ltd. Sperm bundles were isolated from the testes of 2‐3‐day‐old males for immunohistochemistry (IHC) analysis. The bursa copulatrix and spermatheca of 1‐day‐old virgin female adults were used for IHC analysis. The contents were isolated from the spermatophore 1 h after mating for IHC analysis. The testes and spermatophores were dissected and gently torn to release sperm into 1 mL 4% paraformaldehyde, then fixed at room temperature for 1 h. The bursa copulatrix and spermatheca were fixed overnight in 1 mL 4% formaldehyde. Samples were washed four times with 400 µL PAT_3_ buffer (PBS with 0.1% Triton X‐100 and 3% BSA), for 10 m each wash. Next, sperm bundles were incubated in 400 µL of 3% normal goat serum (NGS) at room temperature for 1 h. Primary antibody (anti‐LTNAT, 1:200) was added and incubated for 48 h at 4°C. After four washes with PAT_3_ buffer, each lasting 10 m, samples were incubated with the secondary antibody (donkey anti‐mouse IgG conjugated to Alexa Fluor 555, 1:500) at room temperature for 1 h. Following four washes with 400 µL PAT3, the sperm bundle samples were suspended in PBS. A 10 µL aliquot of the sperm suspension was mounted on a microscope slide and covered with a 20 mm × 20 mm coverslip. The bursa copulatrix and spermatheca samples were mounted in Vectorshield (Vector Laboratory). Fluorescence images were captured using a Zeiss LSM 980 confocal microscope equipped with Airyscan 2 technology. All images were processed with ImageJ software.

### 4.11 Scanning Electron Microscopy (SEM)

Sperm bundles were collected from the testes of a 1‐day‐old male, and sperm were also obtained from the female bursa copulatrix 1 h after mating. Sperm isolated from the testes of 8 males or 5 spermatophores were mixed in one tube as one biological replicate. A total of two biological replicates were used in the SEM experiment. Samples were fixed overnight at 4°C in 4% glutaraldehyde, followed by three washes with phosphate buffer (15 min each). Dehydration was performed using a graded ethanol series. Subsequently, samples were exchanged with tert‐butanol and freeze‐dried using a vacuum freeze dryer. The dried specimens were coated with a 10 nm gold film using an ion sputter coater. Imaging was conducted with a Hitachi Regulus 8100 field‐emission scanning electron microscope.

### 4.12 Transmission Electron Microscopy (TEM)

Sperm bundles were dissected from the testes of 1‐day‐old males and fixed overnight at 4°C in 4% glutaraldehyde. Sperm isolated from the testes of 8 males or 5 spermatophores were mixed in one tube as one biological replicate. A total of two biological replicates were used for the TEM assay. After three washes in phosphate buffer (15 min each), samples were post‐fixed in 2% osmium tetroxide for 2 h at room temperature. Following additional washes, samples were dehydrated through a graded ethanol series and exchanged with propylene oxide. The sperm samples were then embedded in epoxy resin. Ultrathin sections (80 nm) were stained with uranyl acetate and lead citrate for 15 m each. Images were acquired using a Hitachi HT7800 high‐contrast transmission electron microscope.

### 4.13 Proteomic Analysis

Testes from the same size in 1‐day‐old WT and *LTNAT^−/−^* male adults were ground into a fine powder in liquid nitrogen. The tissue powder was lysed in an appropriate volume of SDT buffer (containing 100 mM NaCl) supplemented with 1/100 volume of DTT. The mixture was vortexed thoroughly and subjected to ultrasonication for 5 m on an ice‐water bath. The lysate was then centrifuged at 12,000 × *g* for 15 m at 4°C to collect the supernatant. Protein concentrations were determined using the Bradford Protein Assay Kit (Beyotime). For digestion, the protein samples were dissolved in DB buffer (6 M urea, 100 mM TEAB, pH 8.5) and incubated with trypsin and 100 mM TEAB buffer at 37°C for 4 h. After digestion, formic acid was added to lower the pH below 3. The solution was centrifuged at 12,000 × *g* for 5 m at room temperature. The supernatant was slowly filtered through a C18 desalting column. The column was washed three times with washing buffer (0.1% formic acid, 3% acetonitrile) and eluted with elution buffer (0.1% formic acid, 70% acetonitrile). The eluate was collected and lyophilized. The resulting peptides were resuspended in 10 µL of 0.1% formic acid and analyzed by LC‐MS/MS using a Vanquish Neo UHPLC system coupled with a Thermo Orbitrap Astral mass spectrometer equipped with an Easy‐Spray (ESI) ion source. Ion spray voltage was set at 2.0 kV, and the ion transfer tube temperature was 290°C. Data were acquired in data‐independent acquisition (DIA) mode, with a full scan range of *m/z* 380–980 and MS1 resolution of 240,000 at *m/z* 200. Raw files were analyzed using DIA‐NN software against the NCBI *S. frugiperda* protein database. For peptide and protein identification, the false discovery rate (FDR) was set at 1%. Differentially expressed proteins (DEPs) between experimental and control groups were identified using Student's *t*‐test. Proteins with a *p* < 0.05 and a fold change greater than 1.5 or less than 0.667 were identified as DEPs. The annotation and enrichment methods for GO terms and KEGG pathways are the same as the steps in transcriptome analysis. GO terms and KEGG pathways with a *p* < 0.05 were considered significantly enriched. Gene Set Enrichment Analysis (GSEA) was performed using the gsea‐3.0 software. Pathways or gene sets with *p* < 0.05, adjusted *p* < 0.25, and an absolute normalized enrichment score (|NES|) > 1 were considered significantly enriched. The STRING DB software was used to predict potential protein–protein interactions (http://STRING.embl.de/).

### 4.14 Determination of ATP Content

ATP levels in sperm from the testes of a single male were measured using a luciferase‐based ATP Assay Kit (S0027; Beyotime). To reduce variation in sperm quantity, testes of similar size were selected for analysis. Testes were dissected with forceps and torn open in PBS to release sperm, followed by brief centrifugation to pellet the sperm cells. To each sperm pellet, two stainless steel beads and 100 µL of lysis buffer were added. Samples were homogenized at 120 Hz for 120 s. After homogenization, lysates were centrifuged at 12,000 × *g* for 5 m at 4°C. The supernatant was collected, and luminescence intensity was measured using a PerkinElmer EnVision microplate reader. A series of ATP standard solutions was prepared by serial dilution from a 10 mM ATP stock solution. Luminescence values for each standard were measured to generate a standard curve. ATP concentrations in sperm lysates were calculated based on this standard curve.

### 4.15 Lifespan and Mating Competitiveness Evaluation

Survival curves were plotted to record the lifespan of WT male adults and *LTNAT* mutant male adults. After emergence, the number of dead males was recorded every 24 h. The males were fed with 10% honey water daily. For the mating competitiveness experiment, a 3‐day‐old WT male (with its abdomen painted with green fluorescent paint) and a 3‐day‐old *LTNAT* ^−^ *^/^* ^−^ male were selected to compete for mating with a WT female. After the onset of the dark period, the mating status was observed every 15 m. After mating occurred, the abdomen of the mated male was illuminated with a UV light source to identify the genotypes.

### 4.16 Cage Competition

To assess the impact of releasing a large number of *LTNAT* ^−^ *^/^* ^−^ males on the fertility of WT females, a mating competition assay was performed in insect‐rearing cages (20 × 20 × 20 cm). Adults aged 2–3 days were used in all trials. Previous research results showed that introducing sterile males and WT males into the cage at a ratio of 5:1 could effectively inhibit the fertility of WT female adults [19]. In this study, we adopted this release ratio. In the experimental group, *LTNAT* ^−^ *^/^* ^−^ males and WT males were introduced at a 5:1 ratio, along with 12 WT virgin females. The number of offspring hatched within 7 days was recorded. On day 4, all adults were moved to a new cage to facilitate progeny collection and counting. In the control group, only WT males were added, with the same number of females.

### 4.17 Statistical Analysis

All statistical analyses were conducted using the GraphPad Prism 8 software package (GraphPad, San Diego, CA, USA). Data were first tested for normality. For datasets with a normal distribution, parametric tests were employed to compare groups. For datasets that were not normally distributed, non‐parametric tests were used. The sample sizes and specific statistical tests are indicated in the respective figure legends. All data are expressed as mean ± SEM.

## Author Contributions

S.‐F.W. and H.S. designed research; H.S., P.‐Y.H., and Z.‐R.Z. performed research; H.S., P.‐Y.H., Z.‐R.Z., and C.‐F.G. analyzed data; H.S., S.‐R.P., and S.‐F.W. wrote the paper.

## Conflicts of Interest

The authors declare no conflicts of interest.

## Supporting information




**Supporting File 1**: advs74836‐sup‐0001‐SuppMat.docx.

**Supporting File 2**: advs74836‐sup‐0002‐Movie‐S1.avi.

**Supporting File 3**: advs74836‐sup‐0003‐Movie‐S2.avi.

## Data Availability

All study data are included in the article and/or supporting information. The raw data of transcriptomics and proteomics have been deposited in the National Center for Biotechnology Information. The BioProject accession for the transcriptomics of the Te, MAG, and MCa is no. PRJCA044309. The BioProject accession for the transcriptomics and proteomics of the testes of the *LTNAT* mutants is no. PRJCA044394 and no. PRJCA044400, respectively.
