# Supplementary material for: A Testis‐Specific Aralkylamine N‐Acetyltransferase Regulates Dimorphic Sperm Function and Male Fertility in Moths
Source: Adv Sci (Weinh). 2026 Mar 15;13(30):e16374. doi: 10.1002/advs.202516374 (PMC13248844; doi:10.1002/advs.202516374)
Supplement: Supplementary file 1 — Supporting File 1: advs74836‐sup‐0001‐SuppMat.docx. [file ADVS-13-e16374-s001.docx]

**Supplementary data:**

**A Testis-Specific Aralkylamine N-Acetyltransferase Regulates Dimorphic Sperm Function and Male Fertility in Moths**

Hao Sun^1^, Peng-Yi Huang^1^, Zhi-Ruo Zhang^1^, Cong-Fen Gao^1^, Subba Reddy Palli^2^, Shun-Fan Wu^1^*

^1^State Key Laboratory of Agricultural and Forestry Biosecurity, College of Plant Protection, Nanjing Agricultural University, Nanjing, 210095, China;

^2^Department of Entomology, University of Kentucky, Lexington, KY, USA.

∗ Corresponding author.

*E-mail addresses*: [wusf@njau.edu.cn](mailto:wusf@njau.edu.cn) (S.-F. Wu)


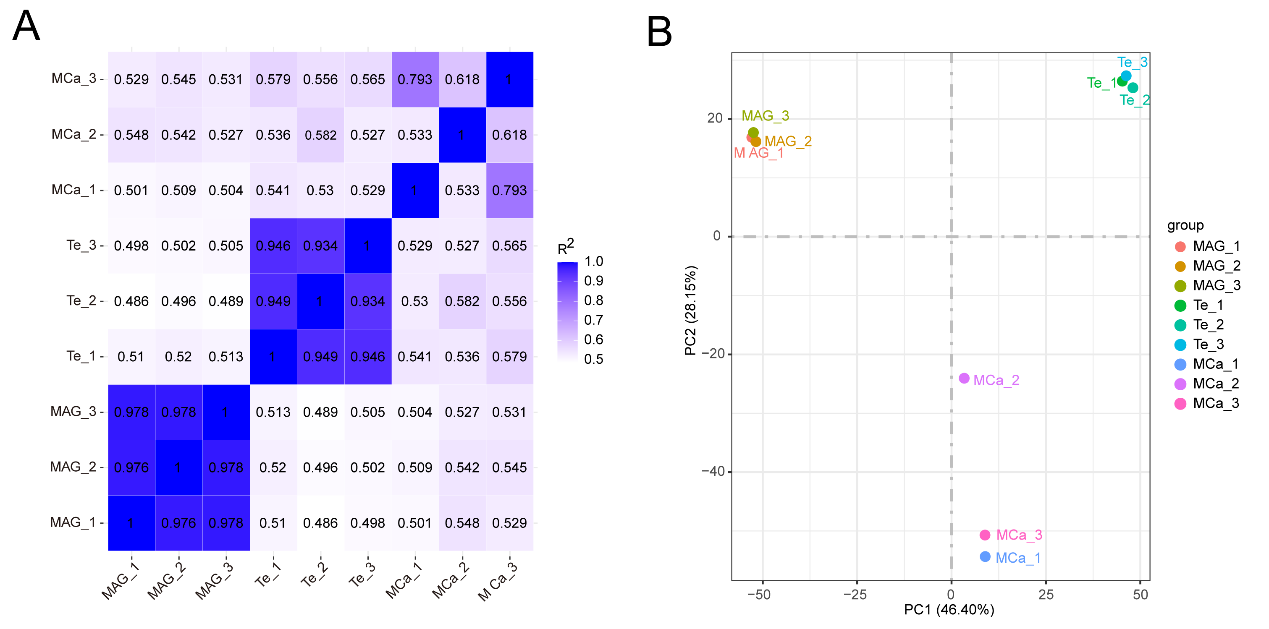


**Figure S1.** Sample correlation analysis. (A) Inter-sample correlation heatmap. (B) Principal component analysis (PCA).


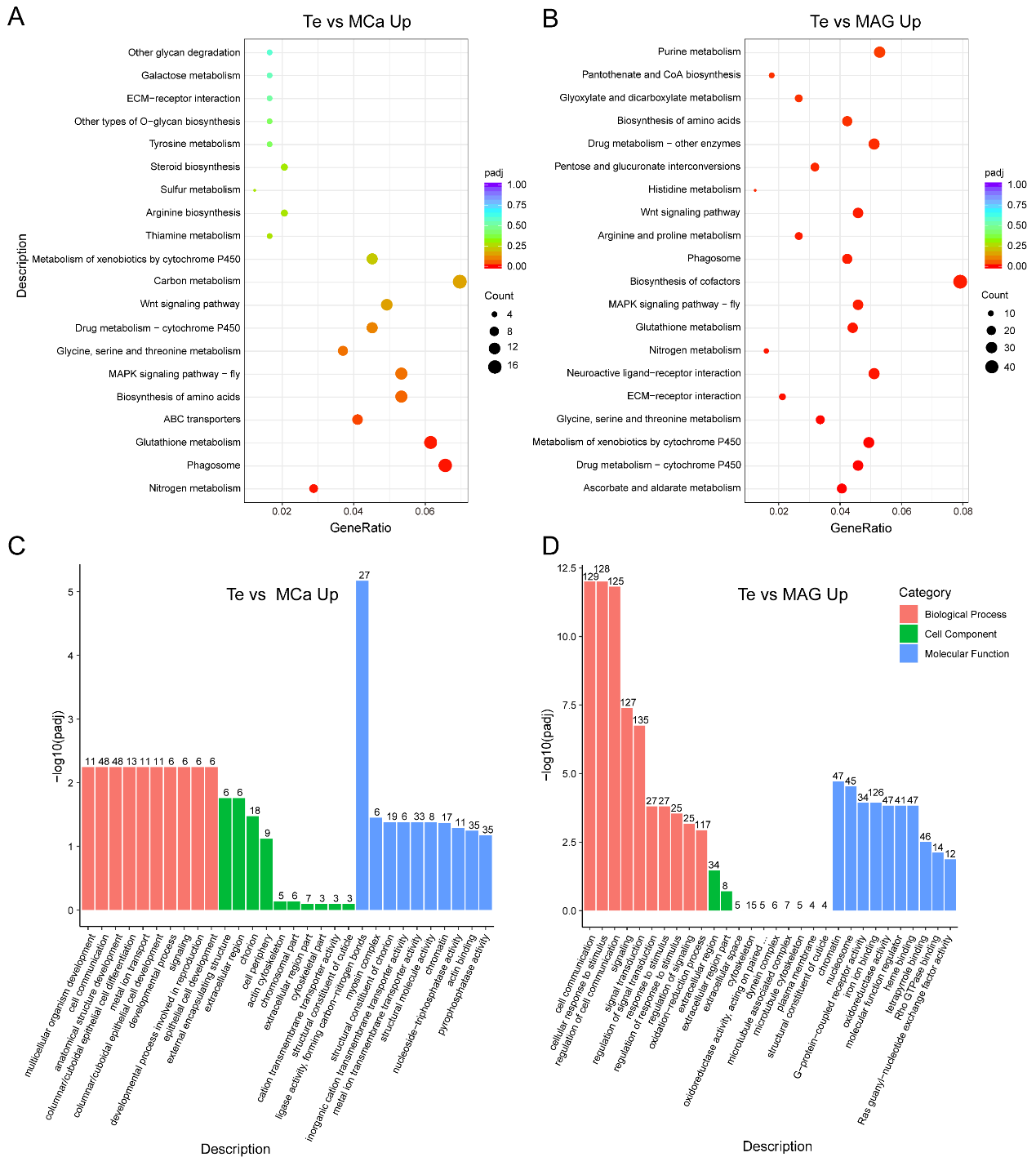


**Figure S2.** KEGG and GO enrichment analysis of testis-enriched genes. (A) KEGG pathway enrichment analysis of upregulated genes in the Te vs MCa. (B) KEGG pathway enrichment analysis of upregulated genes in the Te vs MAG. (C) GO enrichment analysis of upregulated genes in the Te vs MCa. (D) GO enrichment analysis of upregulated genes in the Te vs MAG. Bar colors indicate GO domains: Biological Processes (red), Cellular Components (green), Molecular Functions (blue). Numbers above bars represent the count of upregulated genes associated with each term.


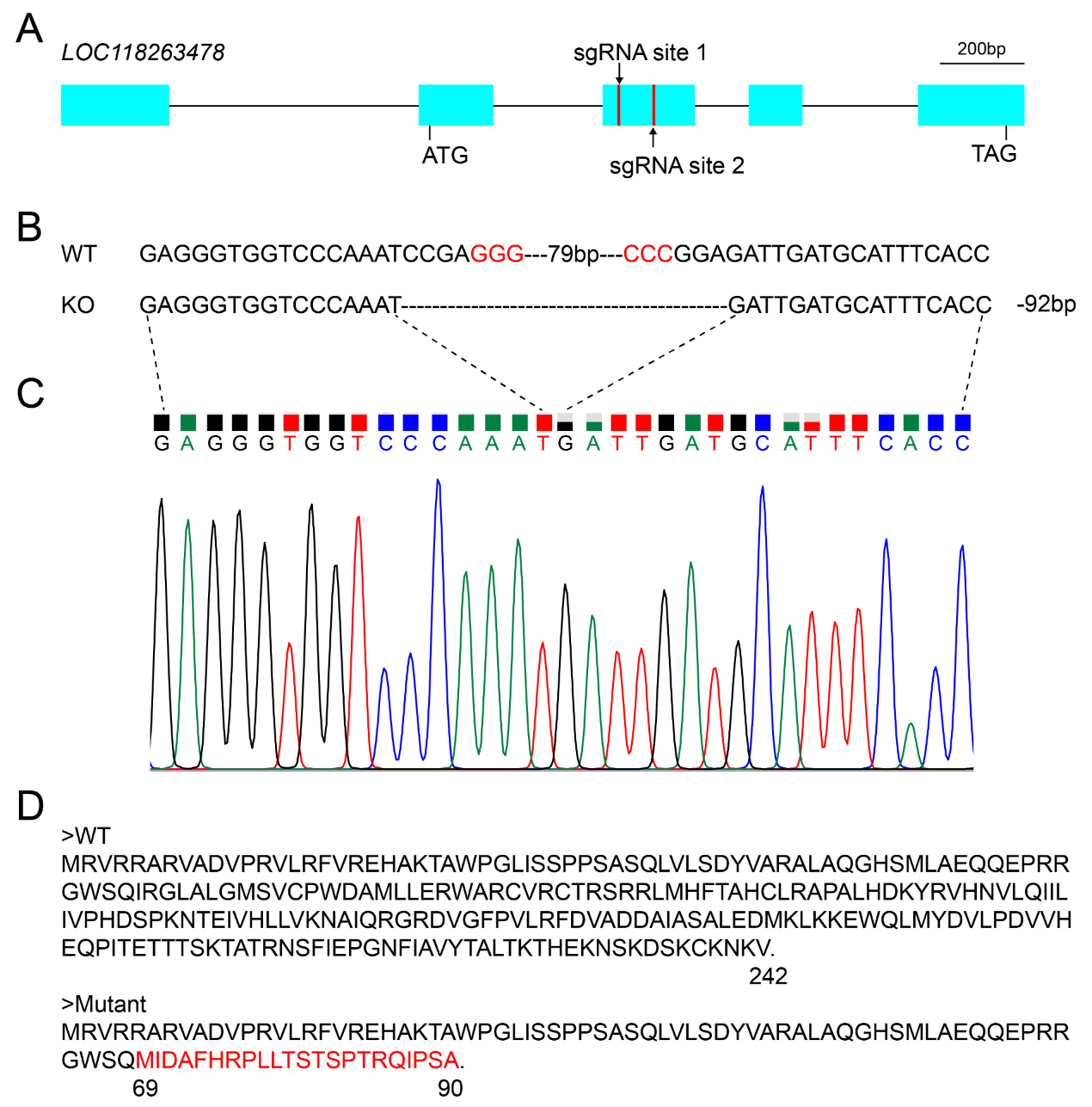


**Figure S3.** CRISPR/Cas9-mediated knockout of *LOC118263478*. (A) Structure of *LOC118263478* and design of single guide RNAs (sgRNAs). The *LOC118263478* consists of 5 exons. Two sgRNAs were designed to target exon 3 of *LOC118263478*. The positions of the start codon, stop codon, and sgRNAs are marked. Scale bar, 200 bp. (B) Construction of a *LOC118263478* gene mutant with a 92-bp deletion. The red bases represent the PAM sequences, and the black bases represent the sgRNA sequences. (C) Sanger chromatogram confirming the 92-bp deletion in homozygous mutants. (D) The base deletion leads to premature termination of the translation of LOC118263478. The numbers represent the lengths of the amino acids, and the red indicates the mis-encoded amino acid sequences.


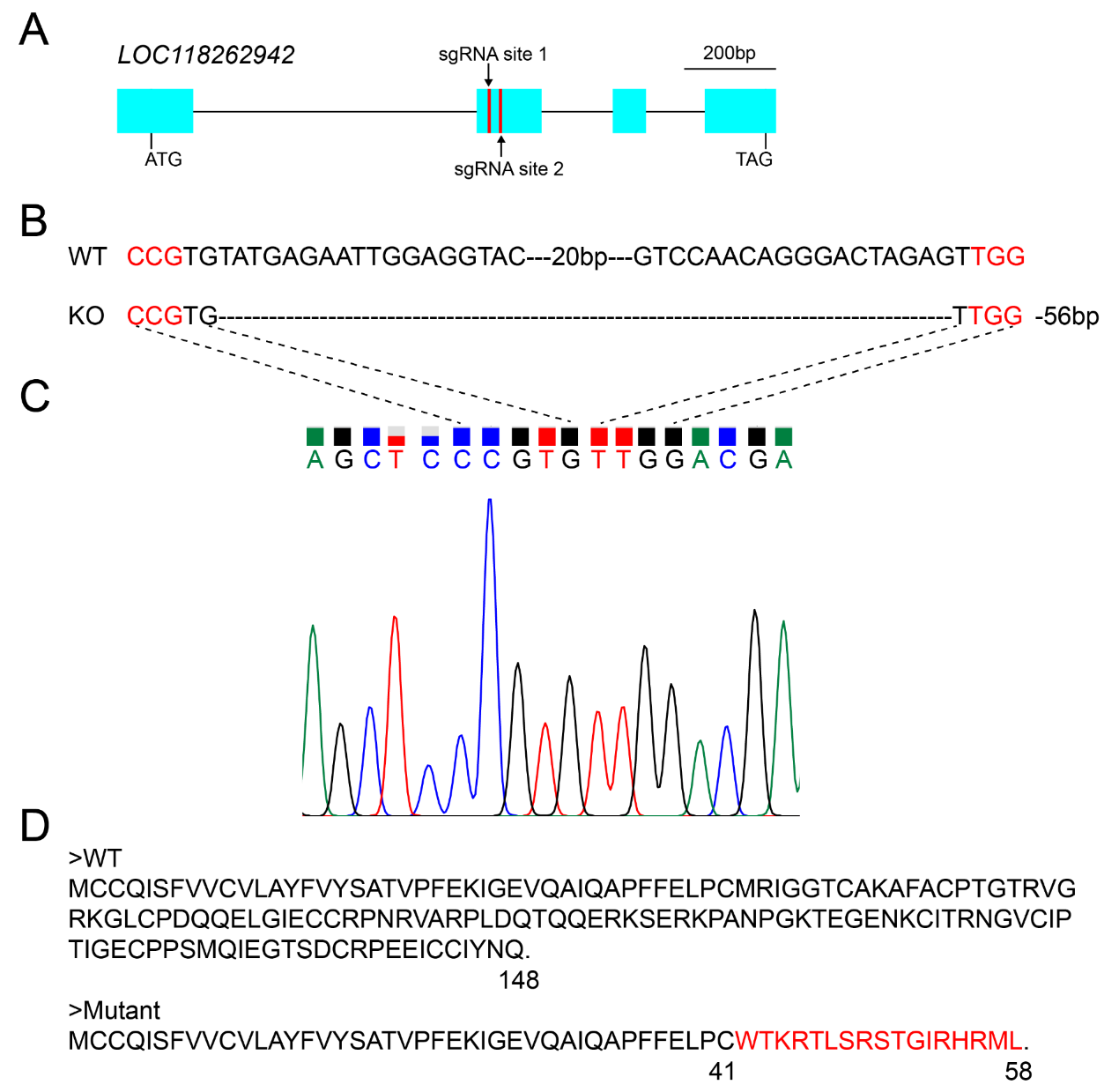


**Figure S4.** CRISPR/Cas9-mediated knockout of *LOC118262942*. (A) Structure of *LOC118262942* and design of sgRNAs. The *LOC118262942* consists of 4 exons. Two sgRNAs were designed to target exon 2 of *LOC118262942*. The positions of the start codon, stop codon, and sgRNAs are marked. Scale bar, 200 bp. (B) Construction of a *LOC118262942* gene mutant with a 56-bp deletion. The red bases represent the PAM sequences, and the black bases represent the sgRNA sequences. (C) Sanger chromatogram confirming the 56-bp deletion in homozygous mutants. (D) The base deletion leads to premature termination of the translation of LOC118262942. The numbers represent the lengths of the amino acids, and the red indicates the mis-encoded amino acid sequences.


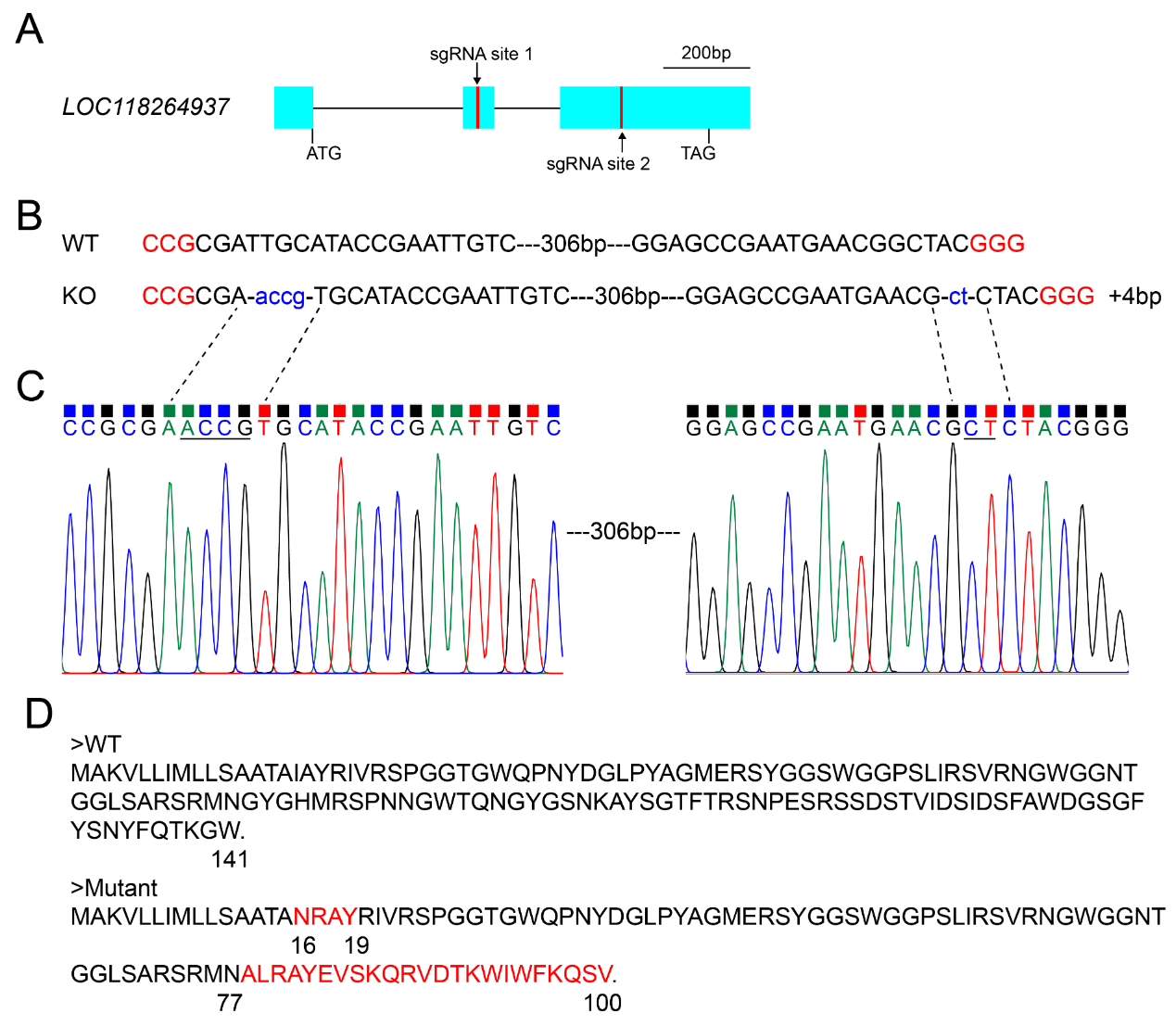


**Figure S5.** CRISPR/Cas9-mediated knockout of *LOC118264937*. (A) Structure of *LOC118264937* and design of sgRNAs. The *LOC118264937* consists of 3 exons. A pair of sgRNAs was designed to target exon 2 and exon 3, respectively. The positions of the start codon, stop codon, and sgRNAs are marked. Scale bar, 200 bp. (B) Construction of a *LOC118264937* gene mutant with a 4-bp insertion. The red bases represent the PAM sequences, the black bases represent the sgRNA sequences, and the blue bases indicate the inserted base sequence. (C) Sanger chromatogram confirming the 4-bp insertion in homozygous mutants. (D) The base insertion leads to premature termination of the translation of LOC118264937. The numbers represent the lengths of the amino acids, and the red indicates the mis-encoded amino acid sequences.


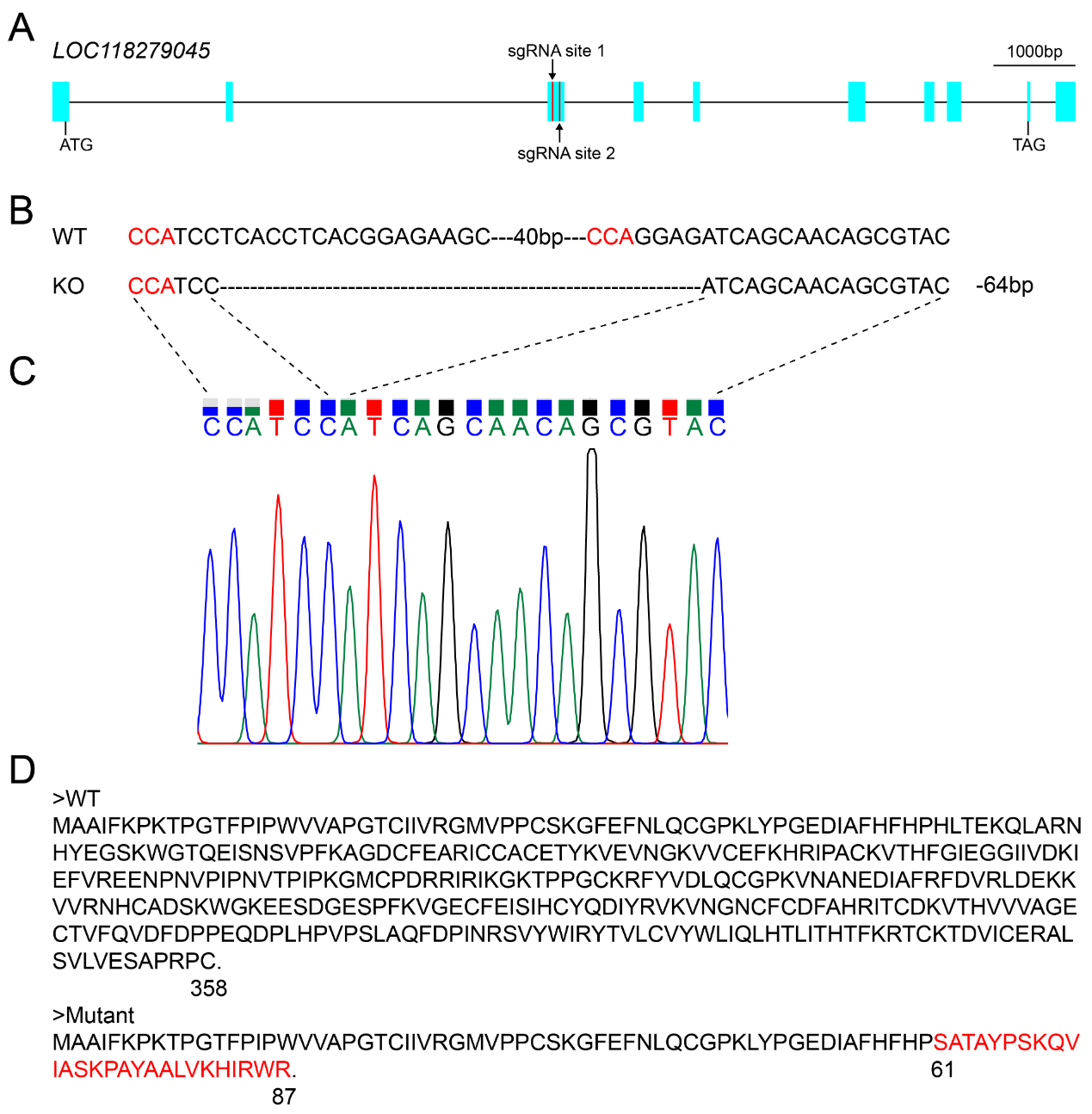


**Figure S6.** CRISPR/Cas9-mediated knockout of *LOC118279045*. (A) Structure of *LOC118279045* and design of sgRNAs. The *LOC118279045* consists of 10 exons. Two sgRNAs were designed to target exon 3 of *LOC118279045*. The positions of the start codon, stop codon, and sgRNAs are marked. Scale bar, 1000 bp. (B) Construction of a *LOC118279045* gene mutant with a 64-bp deletion. The red bases represent the PAM sequences, and the black bases represent the sgRNA sequences. (C) Sanger chromatogram confirming the 64-bp deletion in homozygous mutants. (D) The base deletion leads to premature termination of the translation of LOC118279045. The numbers represent the lengths of the amino acids, and the red indicates the mis-encoded amino acid sequences.


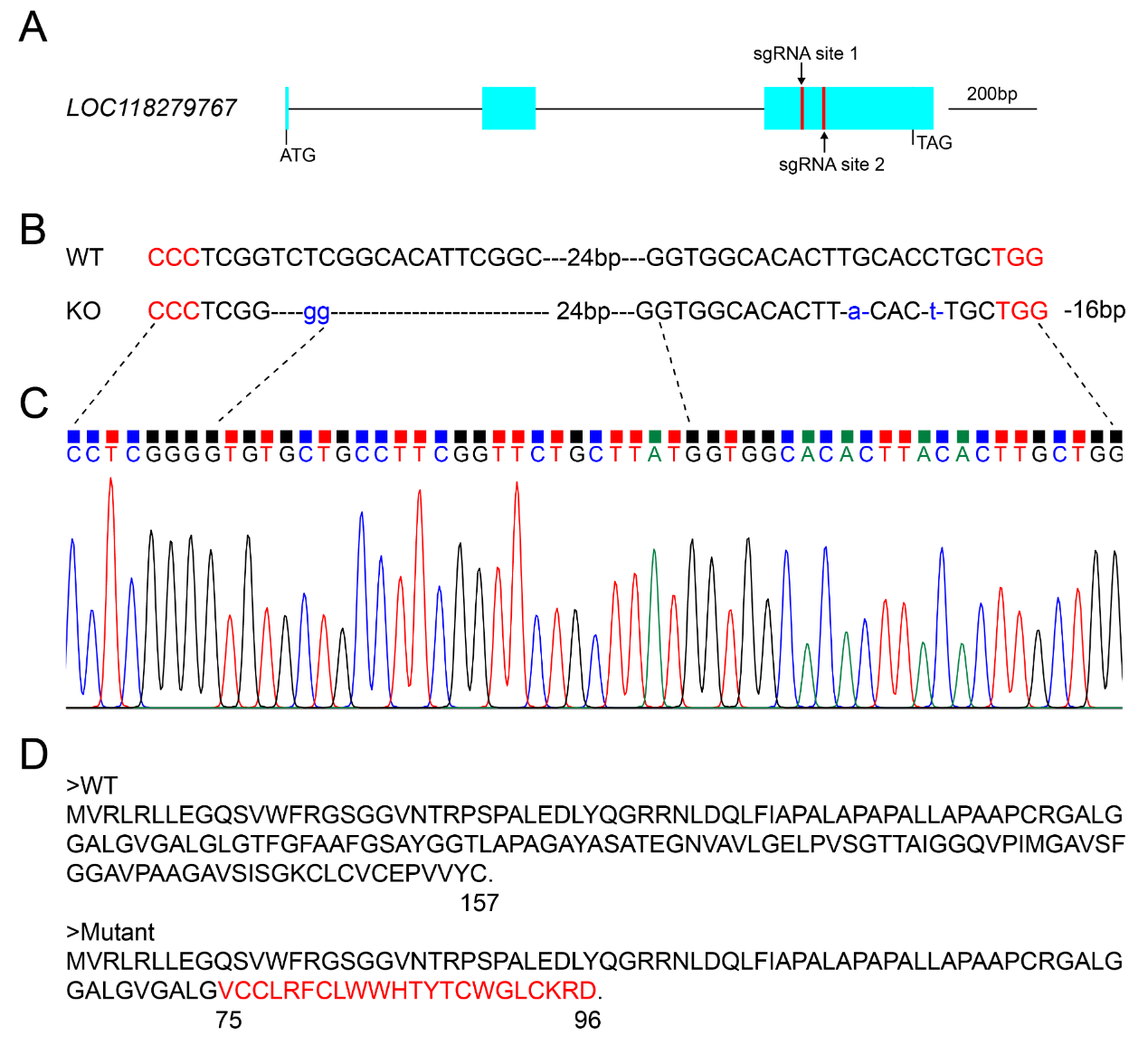


**Figure S7.** CRISPR/Cas9-mediated knockout of *LOC118279767*. (A) Structure of *LOC118279767* and design of sgRNAs. The *LOC118279767* consists of 3 exons. Two sgRNAs were designed to target exon 3 of *LOC118279767*. The positions of the start codon, stop codon, and sgRNAs are marked. Scale bar, 200 bp. (B) Construction of a *LOC118279767* gene mutant with a 16-bp deletion. The red bases represent the PAM sequences, the black bases represent the sgRNA sequences, and the blue bases indicate the inserted base sequence. (C) Sanger chromatogram confirming the 16-bp deletion in homozygous mutants. (D) The base deletion leads to premature termination of the translation of LOC118279767. The numbers represent the lengths of the amino acids, and the red indicates the mis-encoded amino acid sequences.


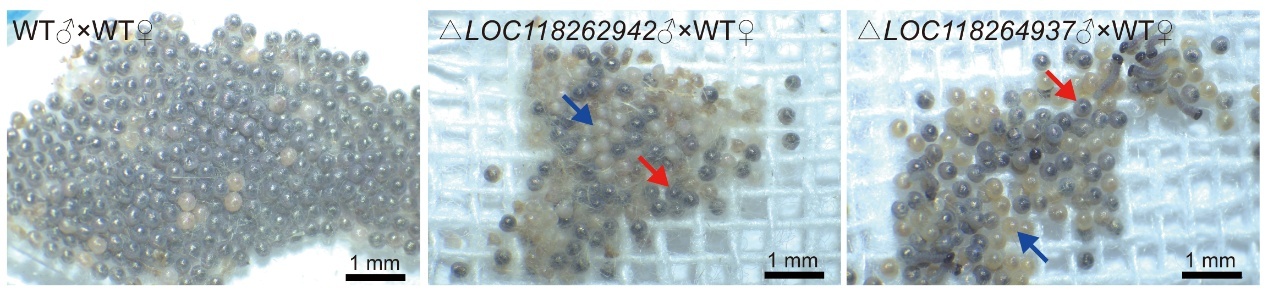


**Figure S8**. Mutations in *LOC118262942* and *LOC118264937* impair male fertility. The proportion of unhatched eggs increases compared with the control group. The blue arrow points to the unhatched eggs, and the red arrow points to the fully developed eggs.

**
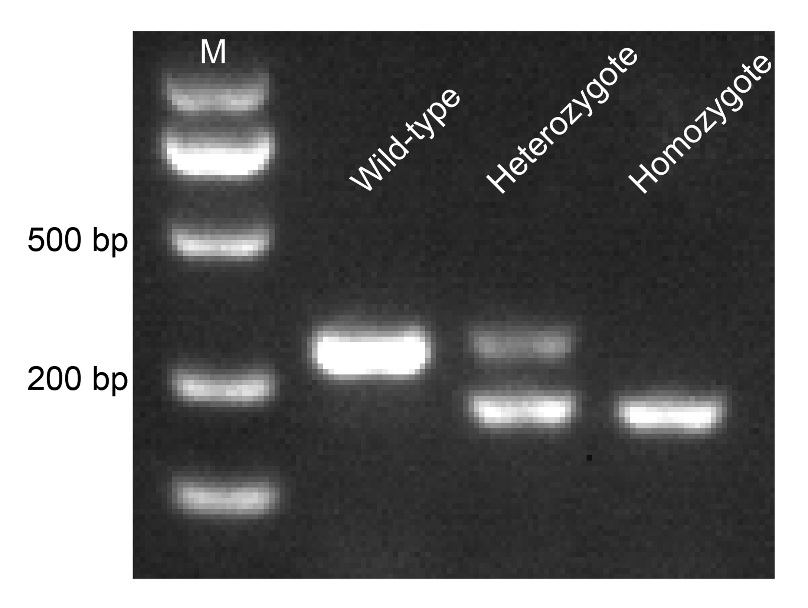
**

**Figure S9.** Genotyping of *LOC118263478* mutation. M: 2000 bp DNA size marker.

**
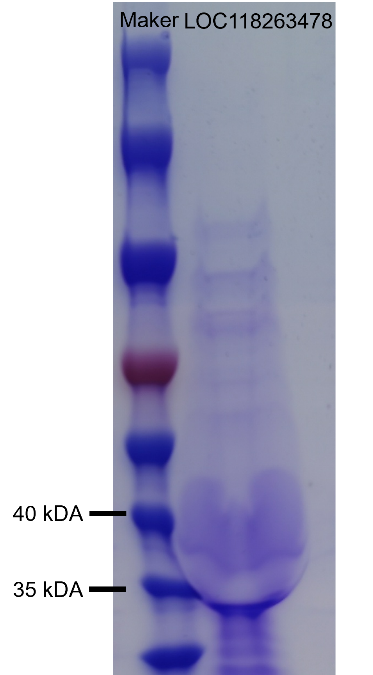
**

**Figure S10**. Ni-NTA-enriched recombinant proteins on SDS-polyacrylamide gel. The purified LOC118263478 and 180 kDa protein Maker were electrophoresed in a 4%-20% SDS-polyacrylamide gel. The predicted molecular weight of the protein encoded by LOC118263478 is approximately 27.5 kDa. The molecular weight of the recombinant LOC118263478 protein is close to 35 kDa. kDa: kilodalton.

**
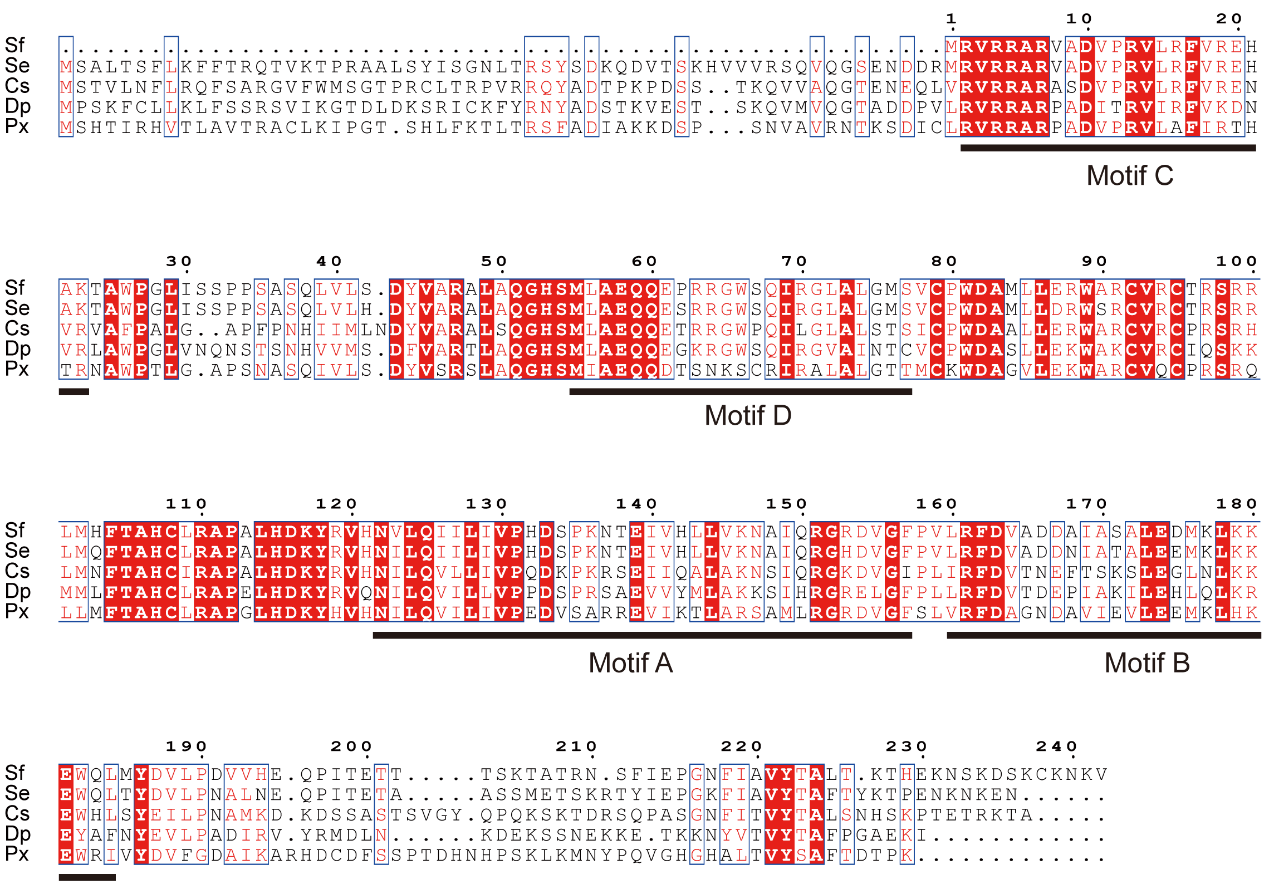
**

**Figure S11.** LOC118263478 homologs are highly conserved among Lepidoptera. Amino acid sequence alignment of LOC118263478 homologs from Spodoptera exigua (Se, CAH0668655.1), Chilo suppressalis (Cs, CAH0663851.1), *Danaus Plexippus* (Dp, XP_032512336.2), and *Papilio Xuthus* (Px, XP_013161602.1). Red backgrounds with white letters mark completely identical residues, representing the highest level of conservation; white backgrounds with red letters mark residues with similar properties, representing secondary conservation. The positions of the motifs are marked below the alignment.


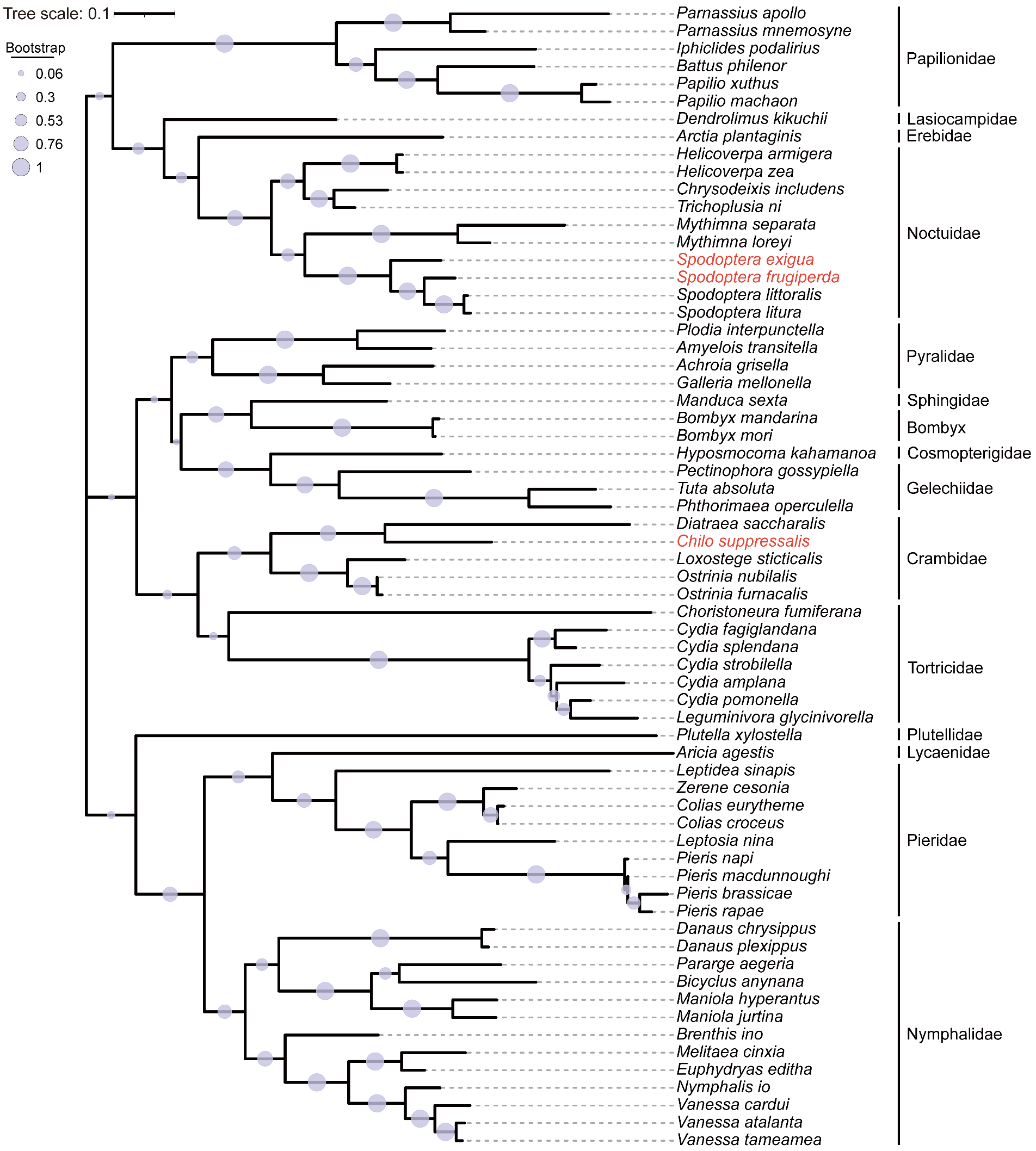


**Figure S12. Phylogenetic analysis of LOC118263478 homologs in Lepidoptera.** The phylogenetic tree was constructed using the maximum likelihood method in MEGA X software based on aligned LTNAT protein sequences from representative Lepidoptera species. Sequence alignment was performed using ClustalW. *S. frugiperda*, *Chilo suppressalis*, and *Spodoptera exigua* are marked in bold red. Bootstrap support values (based on 1000 replicates) are indicated at the nodes. The species names and corresponding GenBank accession numbers are provided in Table S3.


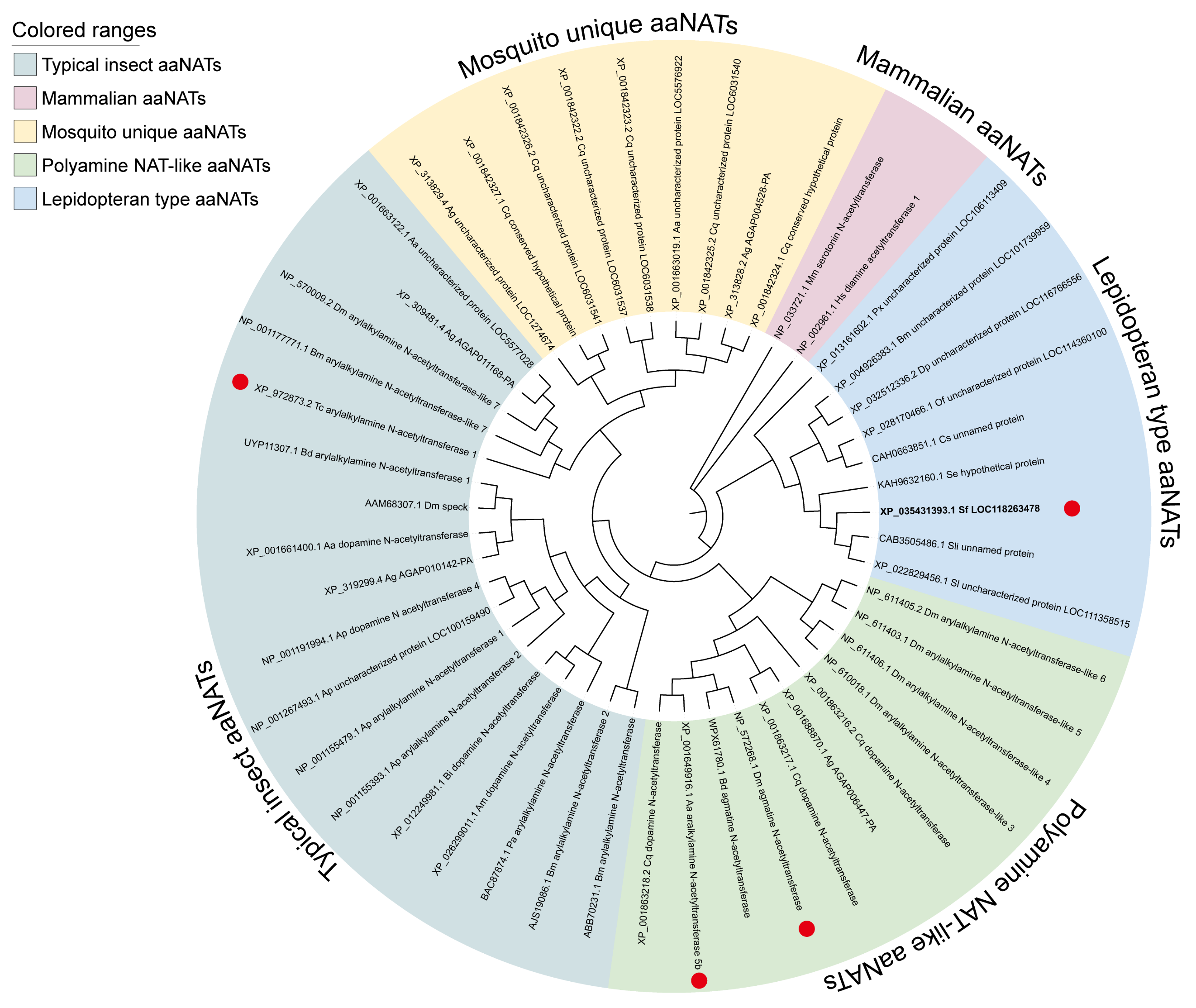


**Figure S13. Evolutionary analysis of** LOC118263478 homologs and aaNATs in mammals and insects. Sequences were aligned using ClustalW. A maximum likelihood phylogenetic analysis was performed using protein sequences of LOC118263478 homologs and aaNATs protein sequences of mammals and other insects. The results revealed that all the protein sequences clustered into five different clades: Mammalian aaNATs, Mosquito unique aaNATs, Polyamine NAT-like aaNATs, Lepidopteran type aaNATs, and Typical insect aaNATs. *S. frugiperda* LOC118263478 is highlighted in bold black. The amino acid sequences used for the multiple sequence alignment in Figure 4B are marked with red circles. The species names were abbreviated accordingly. Bd: *Bactrocera dorsalis*; Dm: *D. melanogaster*; Aa: *A. aegypti*; Ag: *Anopheles gambiae*; Ap: *Acyrthosiphon pisum*; Bi: *Bombus impatiens*; Tc: *T. castaneum*; Cq: *Culex quinquefasciatus*; Cs: *C. suppressalis*; Dp: *Danaus Plexippus*; Px: *Papilio Xuthus*; Se: *S. exigua*; Am: *Apis mellifera*; Pa: *Periplaneta americana*; Hs: *Homo sapiens*; Bm: *B. mori*; Mm: *Mus musculus*; Sl: *Spodoptera litura*; Sli: *Spodoptera littoralis*; Of: Ostrinia furnacalis. The species names and corresponding GenBank accession numbers are marked on the figure.


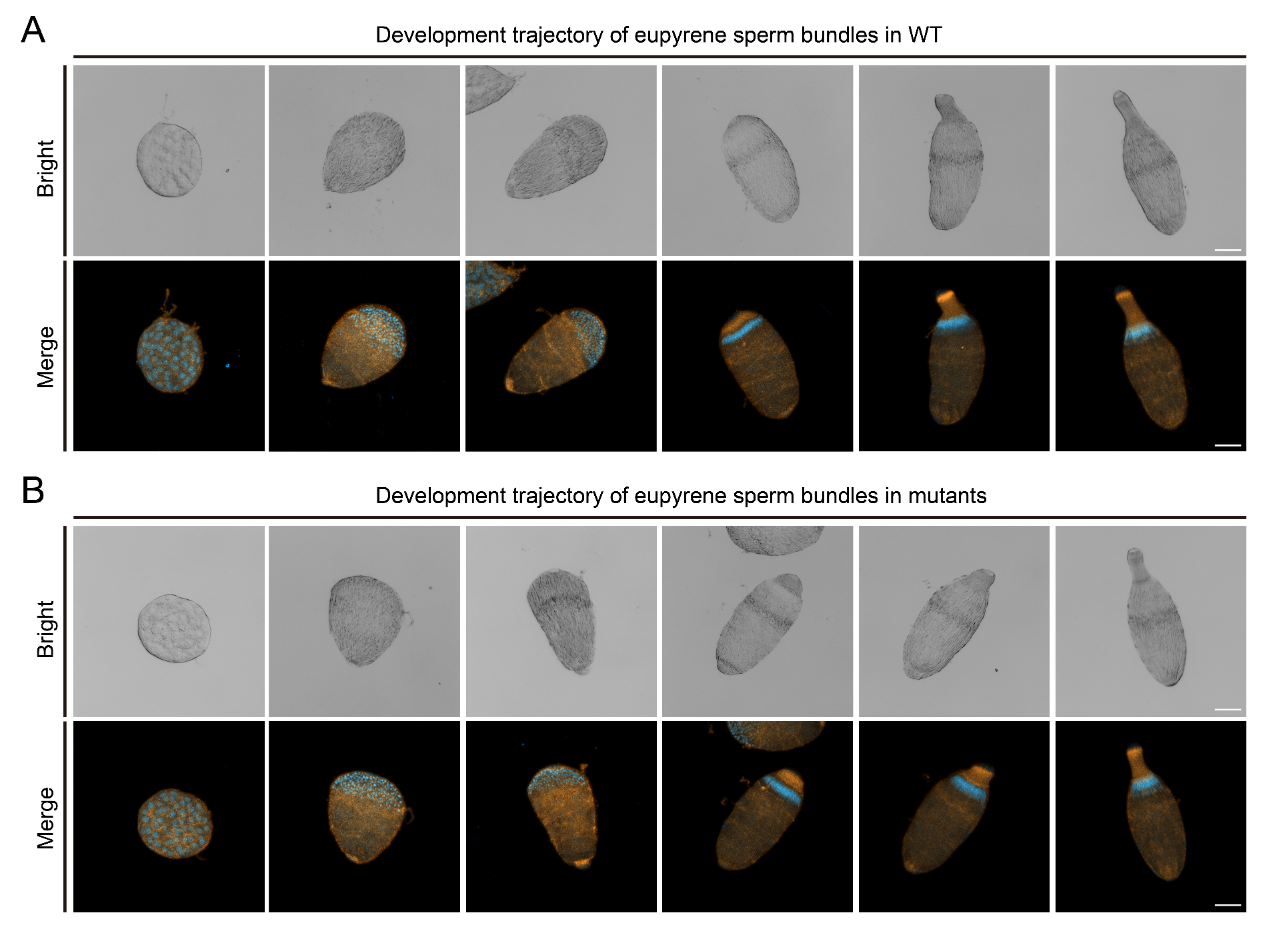


**Figure S14.** *LTNAT* deficiency does not impair early development of eupyrene sperm bundles. (A) Fluorescence staining of eupyrene sperm bundles in testes from WT at L6D4. (B) Fluorescence staining of eupyrene sperm bundles in testes from *LTNAT^-/-^* at L6D4. Nuclei: DAPI (blue); filamentous actin: TRITC-phalloidin (red). Scale bar: 20 μm.


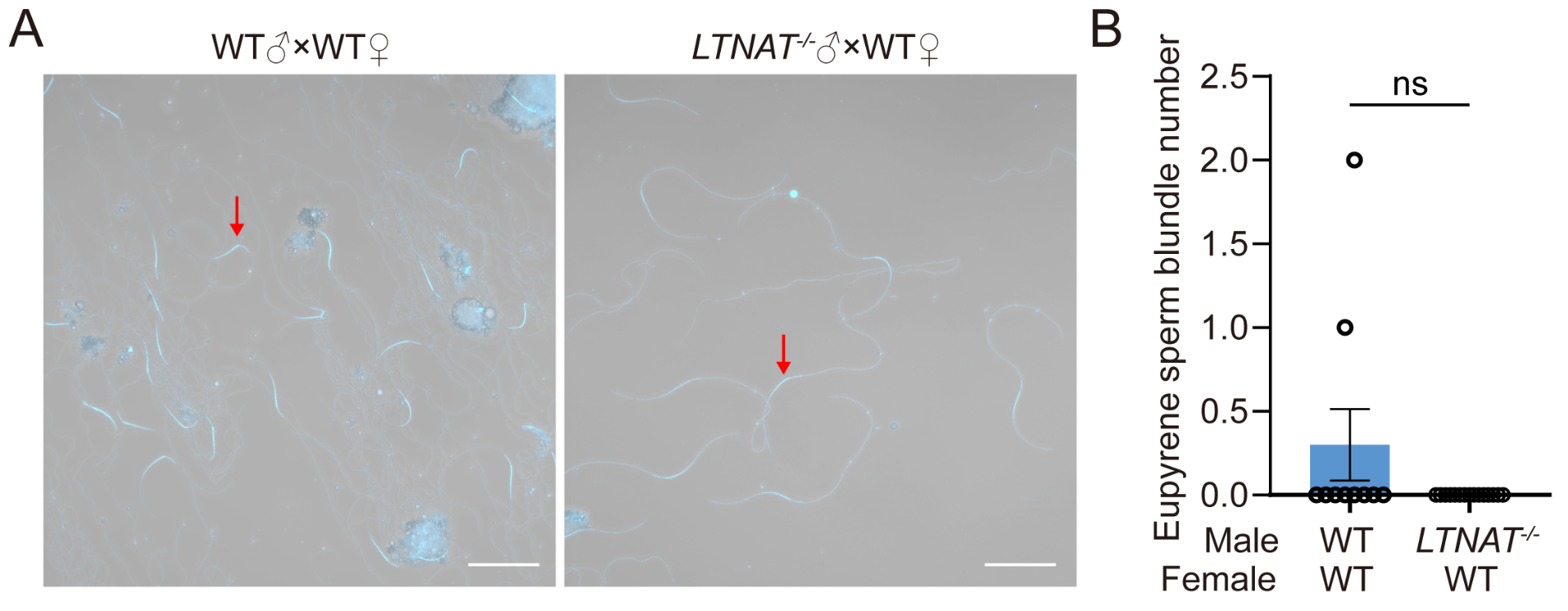


**Figure S15. *LTNAT* disruption did not affect the dissociation of eupyrene sperm bundles. (A) Representative images show the state of eupyrene sperm in the spermatophore. The red arrow indicates the dissociated eupyrene sperm. (B) Statistics of the number of eupyrene sperm bundles in the spermatophore. n = 10-15. Mann-Whitney U test; ns, not significant.**


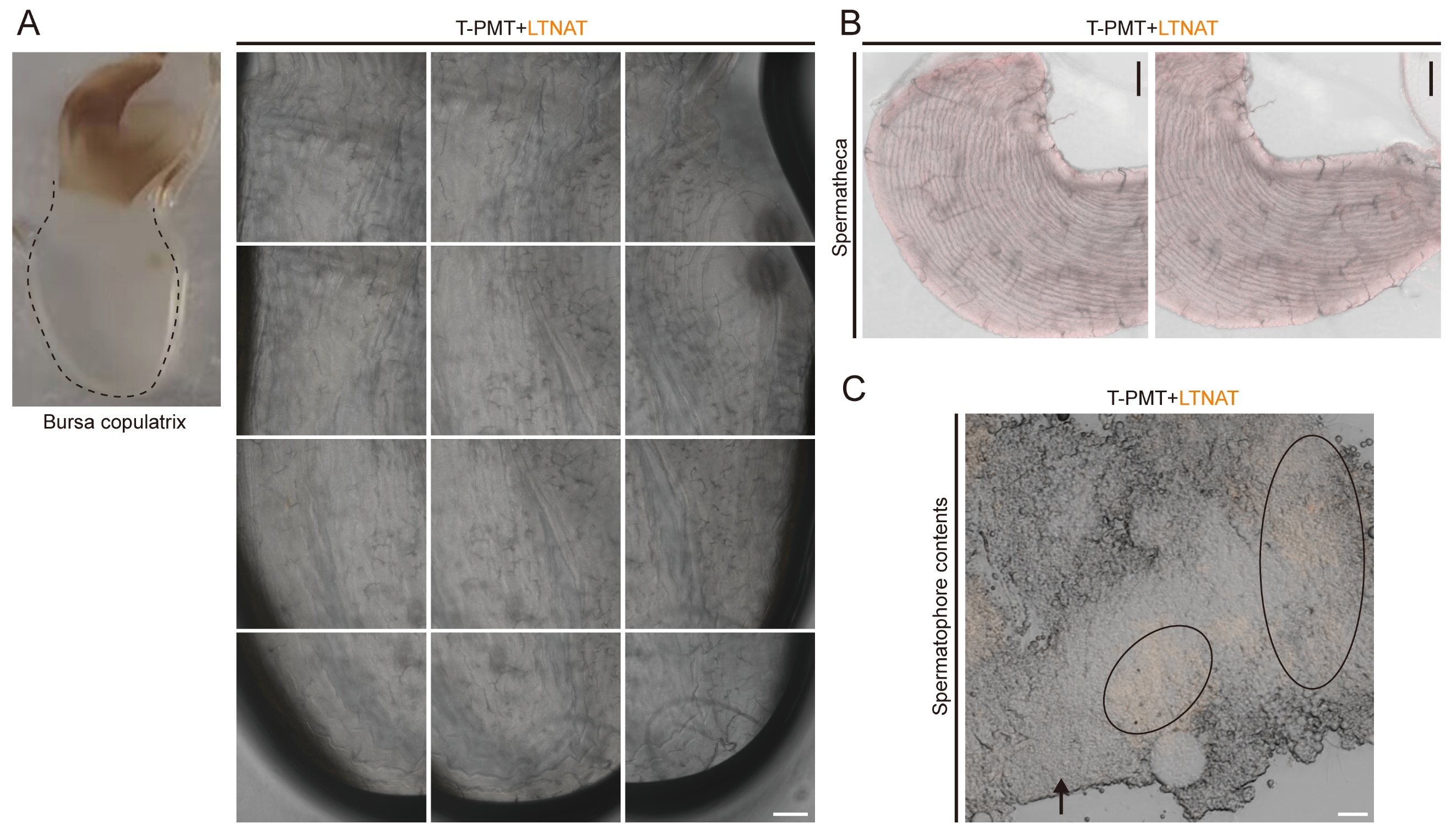


**Figure S16.** LTNAT is not expressed in the reproductive tissues of virgin females and spermatophores. (A) Immunohistochemical analysis of the expression of LTNAT in the virgin bursa copulatrix of virgin females. Scale bar, 100 μm. No obvious LTNAT signal was detected. (B) Immunohistochemical analysis of the expression of LTNAT in the spermatheca of a virgin female. Scale bar, 100 μm. No obvious LTNAT signal was detected. (C) Immunohistochemical analysis of the expression of LTNAT in the spermatophore of a mated female. The circles indicate the stained sperm clusters beneath the protein aggregates in the stained spermatophore contents, and the black arrow indicates the spermatophore contents that do not carry the LTNAT signal. Scale bar, 20 μm.

Movie S1:

Supplementary Movie S1. Motility of apyrene sperm in the bursa copulatrix of females mated to WT males.

Movie S2:

Supplementary Movie S2. Motility of apyrene sperm in the bursa copulatrix of females mated to *LTNAT^-/-^* males.

**
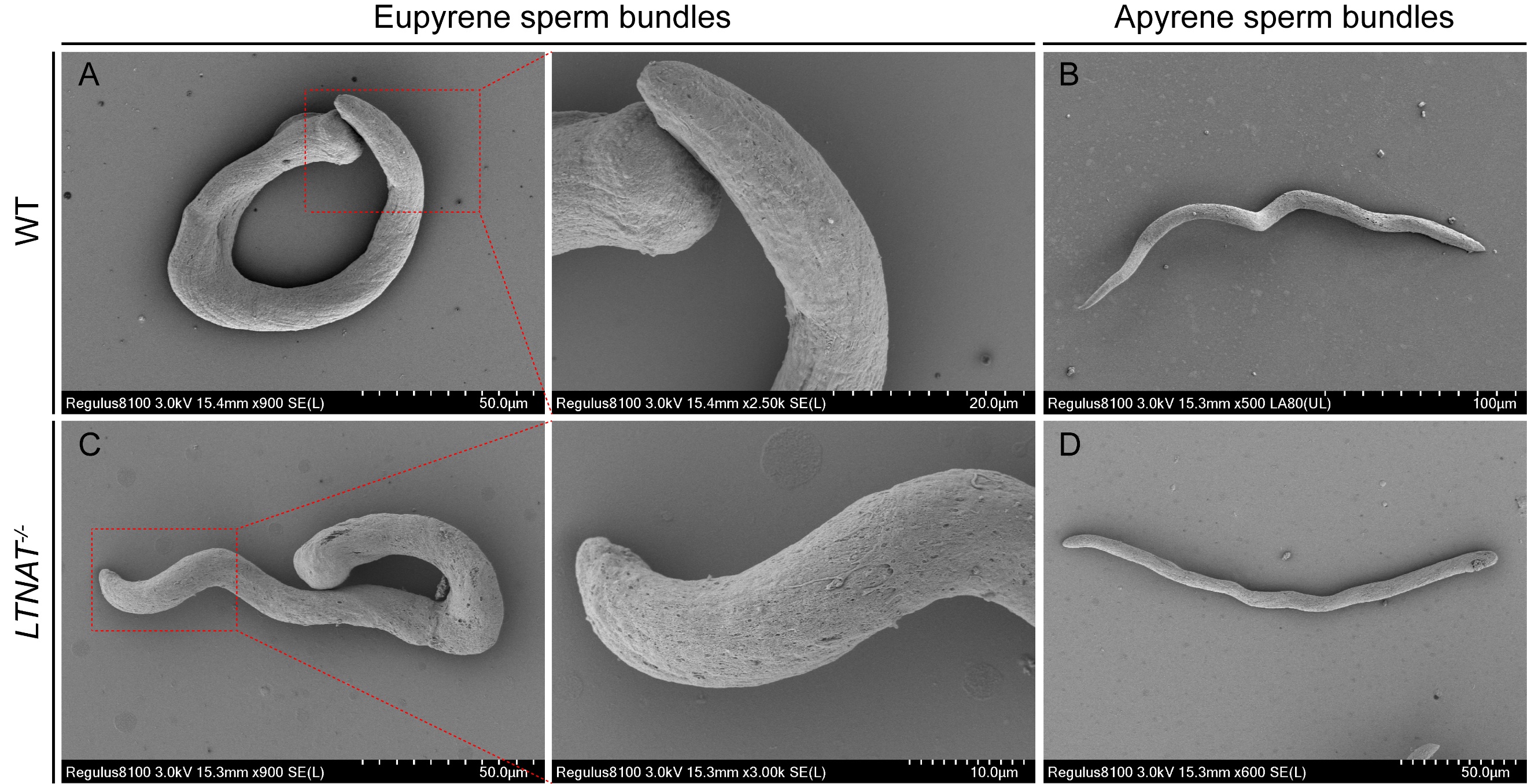
**

**Figure S17.** *LTNAT* deficiency does not disrupt sperm bundle surface morphology. (A) SEM images of eupyrene sperm bundles from WT males. (B) SEM images of apyrene sperm bundles from WT males. (C) SEM images of eupyrene sperm bundles from *LTNAT^-/-^* males. (D) SEM images of apyrene sperm bundles from *LTNAT^-/-^* males. Scale bars are shown in images.


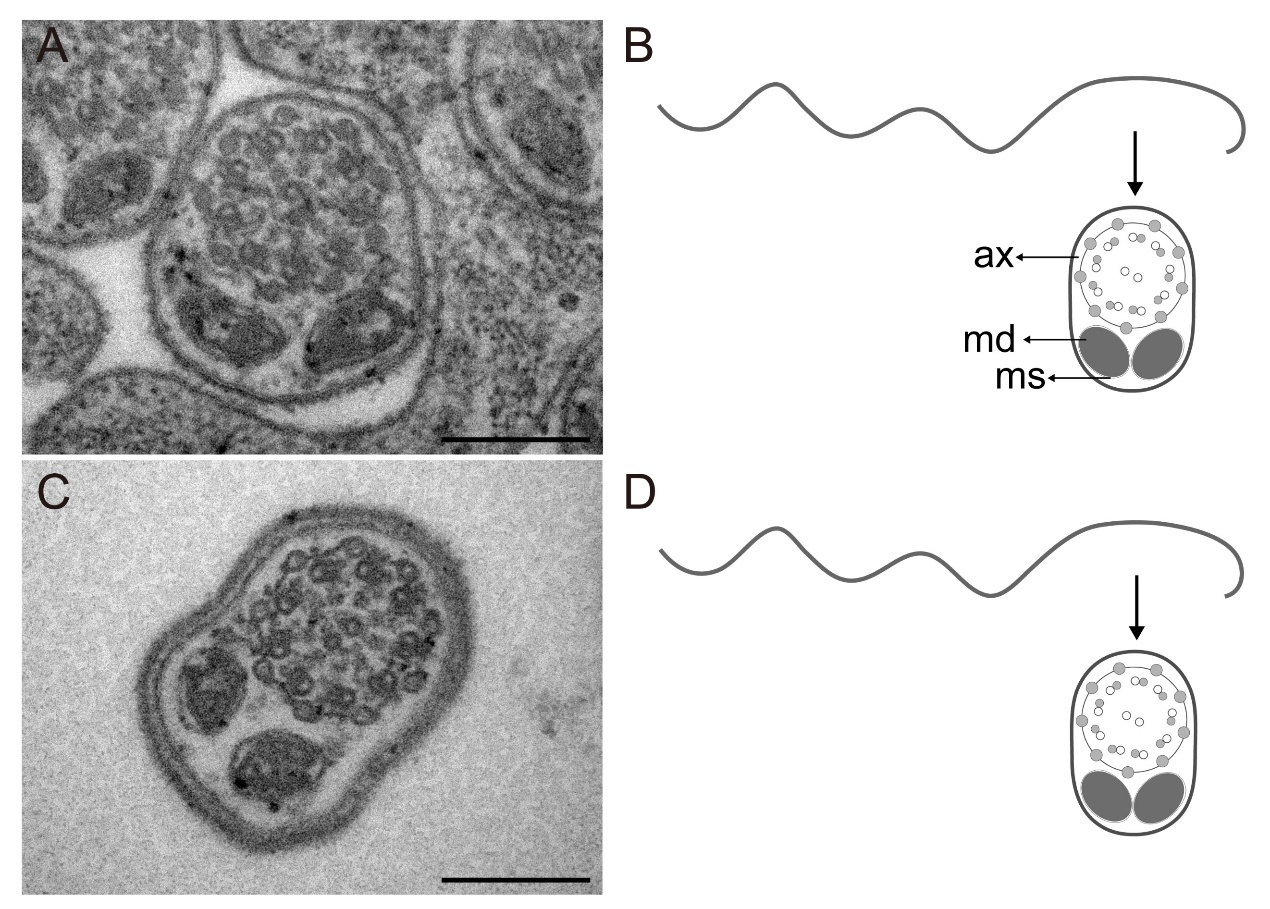


**Figure S18.** Loss of *LTNAT* did not disrupt apyrene sperm flagellar ultrastructure. (A) TEM image of the cross-section of the flagella of WT apyrene sperm. Flagella contain two small MDs with intact ms and the characteristic insect 9+9+2 ax. Scale bars, 200 nm. (B) Cross-sectional structure diagram of the flagella of WT apyrene sperm. (C) TEM images of the cross-section of the flagella of *LTNAT^-/-^* apyrene sperm. Scale bars, 200 nm. (D) Cross-sectional structure diagram of the flagella of *LTNAT^-/-^* apyrene sperm.


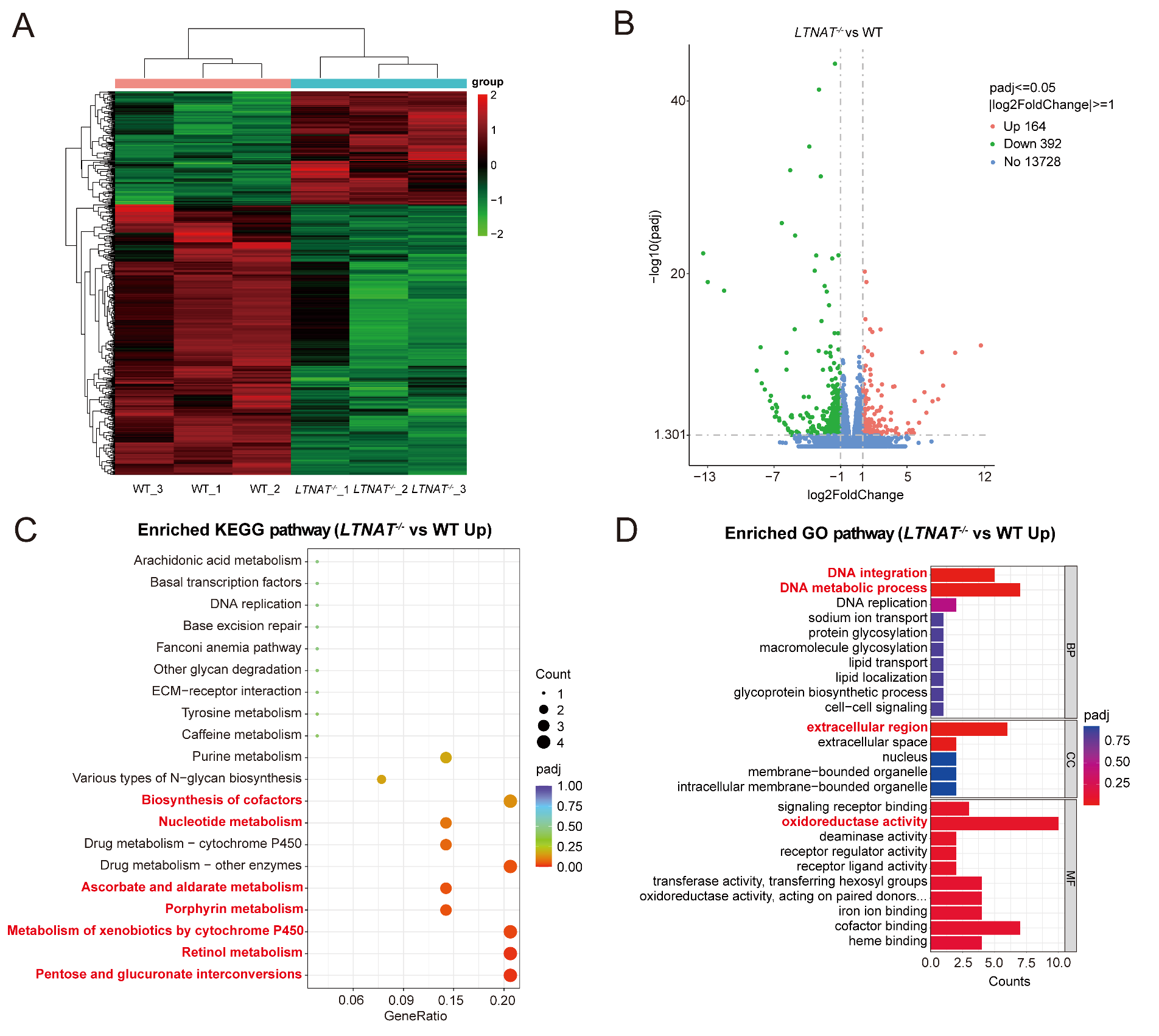


**Figure S19.** Transcriptomic profiling of *LTNAT* deletion reveals altered gene expression and regulatory pathways. (A) mRNA expression heatmap comparing WT and *LTNAT*^-/-^ testes. (B) Volcano plot of differentially expressed genes (DEGs) between WT and *LTNAT*^-/-^ testes. (C) KEGG pathway analysis shows upregulated genes in *LTNAT*^-/-^ testes are enriched in stress response and metabolic remodeling pathways. D) GO analysis reveals significant enrichment of upregulated genes in *LTNAT*^-/-^ testes for: DNA integration, DNA metabolic processes, extracellular region localization, and oxidoreductase activity.


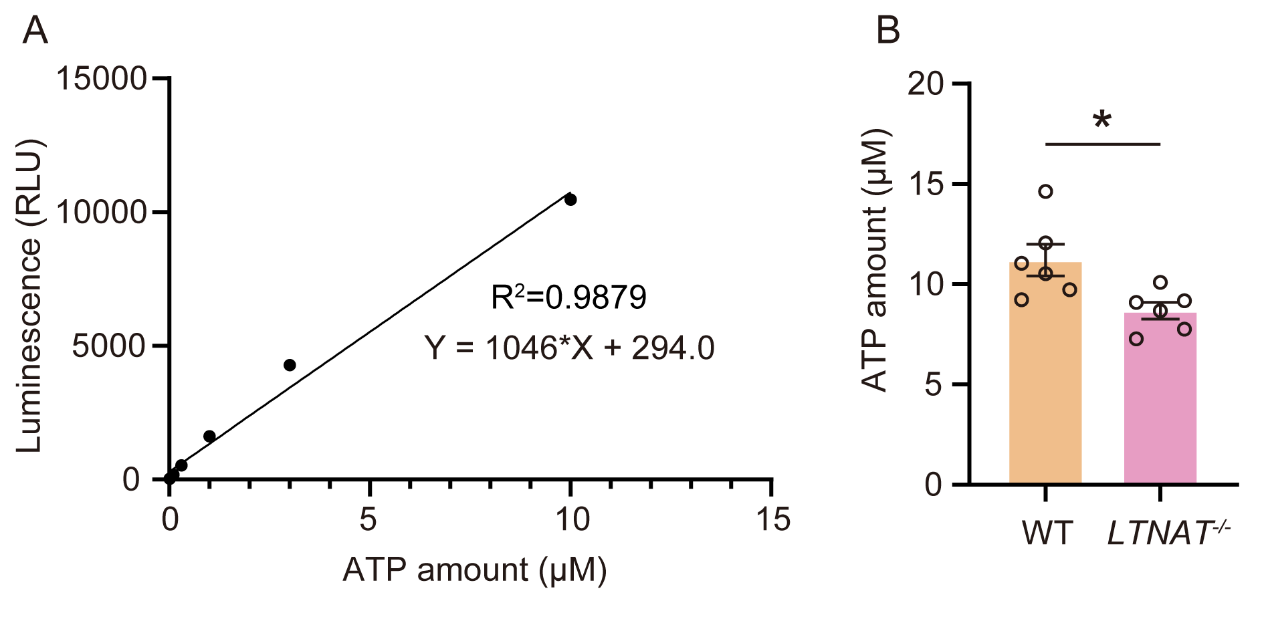


**Figure S20.** *LTNAT* knockout reduces ATP levels in sperm. (A) Plotting standard curves for ATP assays. (B) Quantification of ATP levels in WT and *LTNAT*^-/-^ sperm. n = 6; Student’s *t* test; **P* < 0.05.


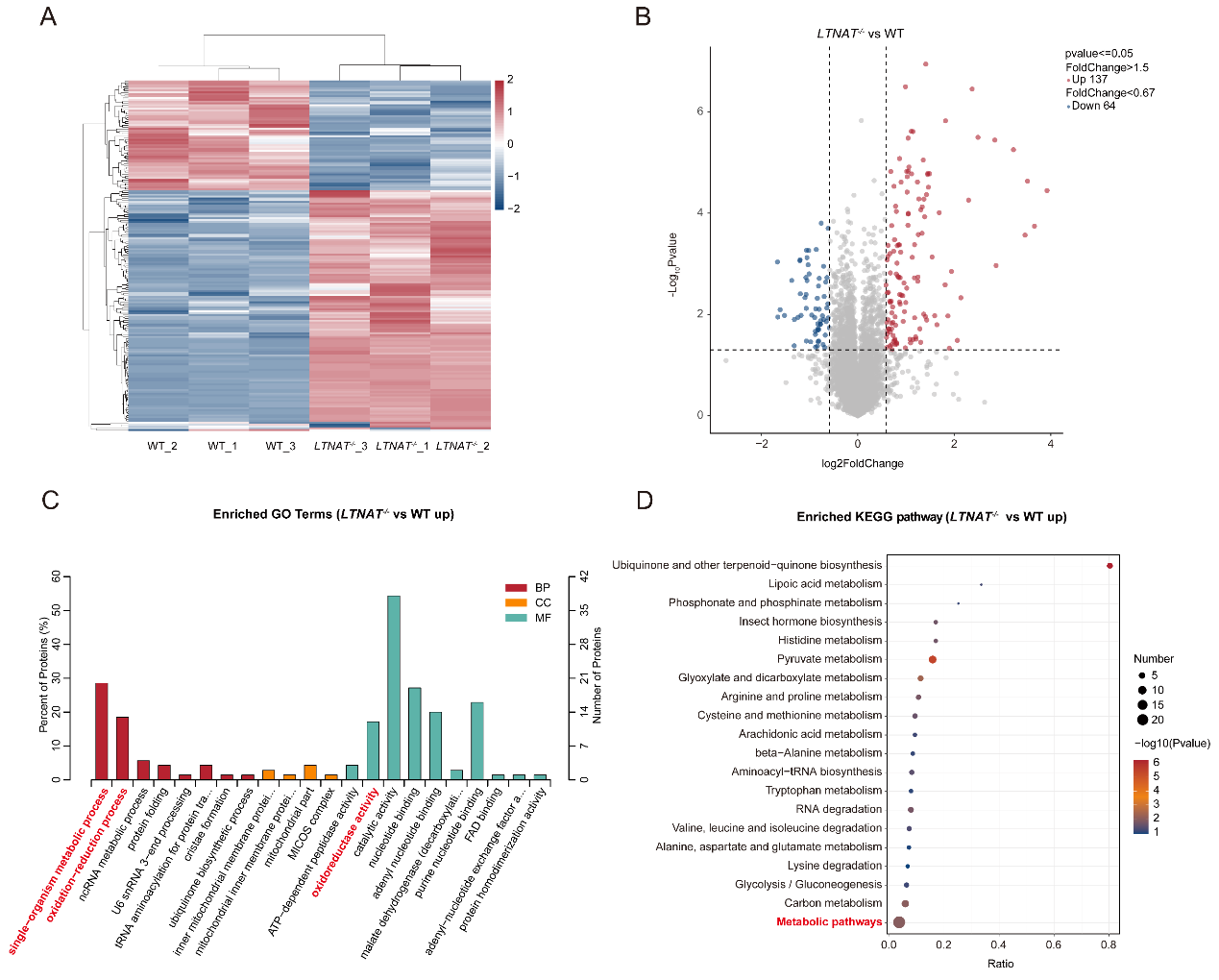


**Figure S21.** Proteomic profiling reveals *LTNAT* knockout disrupts protein expression and metabolic pathways. (A) Heatmap of protein expression changes in WT versus *LTNAT^-/-^* testes. (B) Volcano plot of DEPs in WT versus *LTNAT^-/-^* testes. (C) GO analysis shows significant enrichment of oxidation-reduction processes in WT vs *LTNAT^-/-^* testes. (D) KEGG pathway analysis indicates upregulated proteins in *LTNAT^-/-^* testes associate with metabolic pathways.


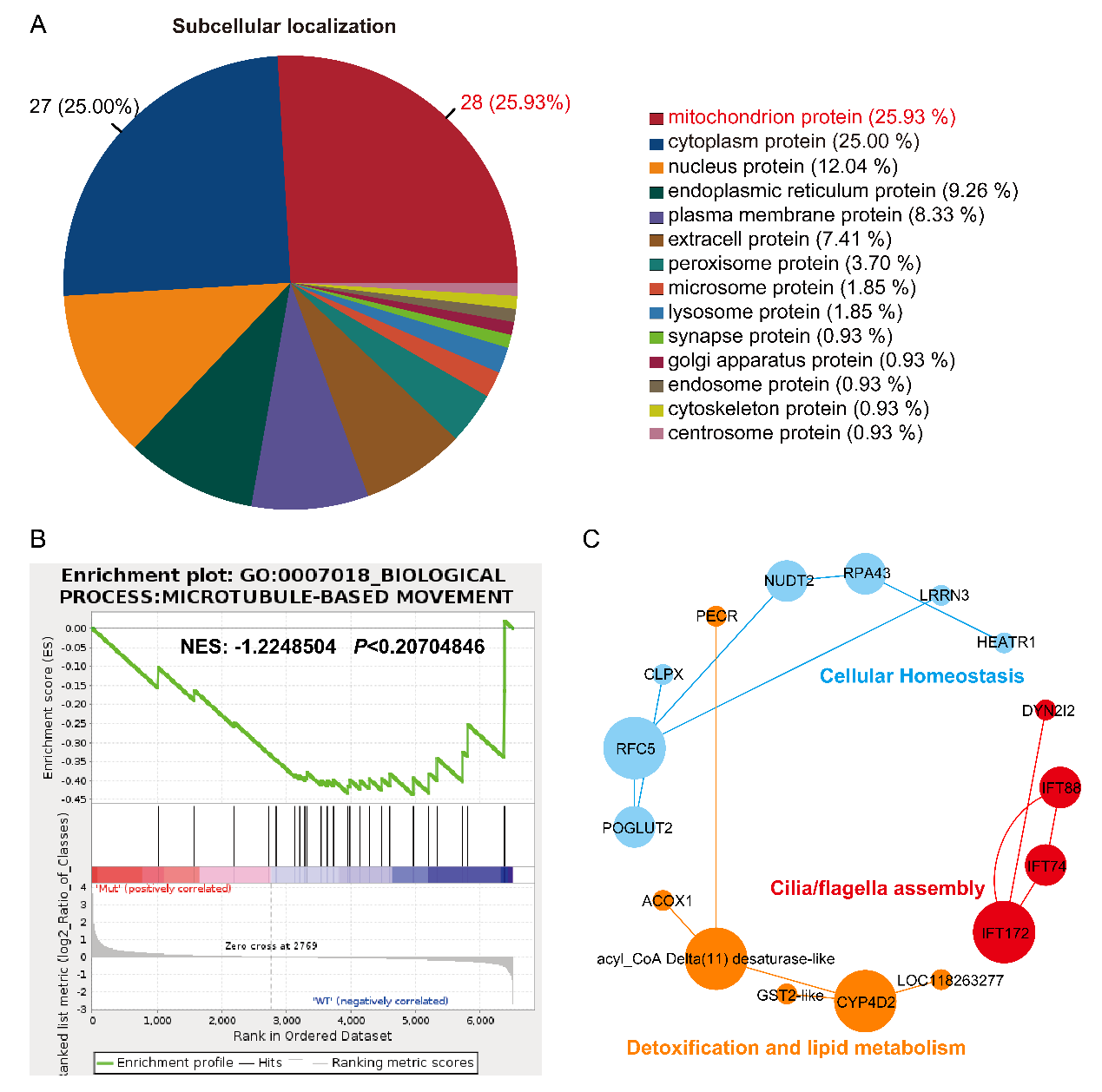


**Figure S22.** *LTNAT* mutation alters the expression of mitochondrial proteins and flagellar/ciliary assembly proteins. (A) Subcellular localization of differentially abundant proteins indicates that proteins altered in *LTNAT^-/-^* vs WT testes predominantly localize to mitochondria (25.93%). (B) GSEA indicates significant enrichment of microtubule-based movement protein sets in WT compared to *LTNAT^-/-^* testes. (C) Interaction network of downregulated proteins in *LTNAT^-/-^* testes. Functionally distinct protein sets are color-coded. Circle size reflects interaction degree (max: 3; min: 1).

**Table S1** The primers used in this study

| Purpose | Primer name | Sequence (5’-3’) |
| --- | --- | --- |
| Gene cloning | 118263478-F | ATCATCAATCCTCAGCTGCC |
|  | 118263478-R | CTGAACAGCCGTCATTAAGC |
|  | 118262942-F | ATCGAGCAGTTTACAACTGG |
|  | 118262942-R | ACAGCATATTTCTTCTGGTC |
|  | 118264937-F | ACAGGACTAGACGTGATCGT |
|  | 118264937-R | GACATCTACCATCCCTTAGT |
|  | 118279767-F | ATGGTGCGATTGAGGTTGTT |
|  | 118279767-R | AGTGGGATCTCAGCAGTAGA |
|  | 118279045-F | ATATTGGCCCTCAAACCTCT |
|  | 118279045-R | GCCAGCATCTCTTCTGTGTT |
|  | CsLTNAT-F | TGTCAACAGTGCTGAATTTC |
|  | CsLTNAT-R | TTAGGCTGTCTTCCTTGTTT |
|  | SeLTNAT-F | ATGTCCGCATTGACAAGTTT |
|  | SeLTNAT-R | GGTGTCTTATATGTGAATGC |
| RT-PCR | 118263478-qF | TCCCAGTGTTGCGGTTCGAT |
|  | 118263478-qR | TCTCGTTGCGGTCTTGCTTG |
|  | 118262942-qF | GGCATAGAATGCTGTAGACC |
|  | 118262942-qR | GCATTTATTTTCGCCCTCCG |
|  | 118264937-qF | GAGGTCTCCAAACAACGGGT |
|  | 118264937-qR | GTGGAGTCTGAGGATCTTGA |
|  | 118279767-qF | TCCGGTACTACTGCTATCGG |
|  | 118279767-qR | GGAGATAGACACGGCTCCAG |
|  | 118279045-qF | AGGAGATCAGCAACAGCGTA |
|  | 118279045-qR | CGATGCCAAAGTGTGTCACC |
|  | CsLTNAT-qF | CCACGACAAGTACAGGGTTC |
|  | CsLTNAT-qR | GTGGGATTCCGACATCCTTC |
|  | SeLTNAT-qF | CCTCATCTCCTCACCACCAT |
|  | SeLTNAT-qR | GACCATCCTCGTCTCGATTC |
|  | RpL18-qF | CGTATCAACCGACCTCCACT |
|  | RpL18-qR | AGGCACCTTGTAGAGCCTCA |
|  | CsActinA1-qF | GTCGCTTCCCAAATTACATC |
|  | CsActinA1-qF | CTCCATATCGTTCCAGTCG |
|  | SeGAPDH-qF | GACAACCACTCATCTATCTTCG |
|  | SeGAPDH-qR | AACATTTATCTCTACAACGCAAC |
| Detect  Mutations | 118263478-MF | CTCGCAGTTCTATGCCTTAG |
|  | 118263478-MR | TGCAGAACATTATGCACTCG |
|  | 118262942-MF | TTCCAGCAATACAAGCACCA |
|  | 118262942-MR | ACGTTGCCGAAGAACTTACT |
|  | 118264937-MF | CTCGGATACTTTTACCAGAT |
|  | 118264937-MR | TAAAGGTACCACTATACGCT |
|  | 118279767-MF | TCATTAAACTGTTGGCTGCG |
|  | 118279767-MR | ATAGCAGTAGTACCGGAGAC |
|  | 118279045-MF | GACTTTGACGAATTGGTTCT |
|  | 118279045-MR | GTAATCTTGGAGCTTACCTT |
|  | CsLTNAT-MF | TACTGCTTTTAGGCTCAGGG |
|  | CsLTNAT-MR | CATCGAGCTCCACTTTCCTT |
|  | SeLTNAT-MF | ATTCTGTTTGCAGGTGCAGG |
|  | SeLTNAT-MR | GCAACAGGTTACAGAAGACC |
| gRNA | 118263478-gF1 | TAATACGACTCACTATAGAGGGTGGTCCCAAATCCGA |
| Synthesis | 118263478-gR1 | TTCTAGCTCTAAAACTCGGATTTGGGACCACCCTC |
|  | 118263478-gF2 | TAATACGACTCACTATAGGTGAAATGCATCAATCTCC |
|  | 118263478-gR2 | TTCTAGCTCTAAAACGGAGATTGATGCATTTCACC |
|  | 118262942-gF1 | TAATACGACTCACTATAGTCCAACAGGGACTAGAGT |
|  | 118262942-gR1 | TTCTAGCTCTAAAACACTCTAGTCCCTGTTGGAC |
|  | 118262942-gF2 | TAATACGACTCACTATAGTACCTCCAATTCTCATACA |
|  | 118262942-gR2 | TTCTAGCTCTAAAACTGTATGAGAATTGGAGGTAC |
|  | 118264937-gF1 | TAATACGACTCACTATAGACAATTCGGTATGCAATCG |
|  | 118264937-gR1 | TTCTAGCTCTAAAACCGATTGCATACCGAATTGTC |
|  | 118264937-gF2 | TAATACGACTCACTATAGGAGCCGAATGAACGGCTAC |
|  | 118264937-gR2 | TTCTAGCTCTAAAACGTAGCCGTTCATTCGGCTCC |
|  | 118279767-gF1 | TAATACGACTCACTATAGCCGAATGTGCCGAGACCGA |
|  | 118279767-gR1 | TTCTAGCTCTAAAACTCGGTCTCGGCACATTCGGC |
|  | 118279767-gF2 | TAATACGACTCACTATAGGTGGCACACTTGCACCTGC |
|  | 118279767-gR2 | TTCTAGCTCTAAAACGCAGGTGCAAGTGTGCCACC |
|  | CsLTNAT-gF1 | TAATACGACTCACTATAGCACGTTCTCTCGCACGAAG |
|  | CsLTNAT-gR1 | TTCTAGCTCTAAAACCTTCGTGCGAGAGAACGTGC |
|  | CsLTNAT-gF2 | TAATACGACTCACTATAGATGATGTGGTTAGGAAACG |
|  | CsLTNAT-gR2 | TTCTAGCTCTAAAACCGTTTCCTAACCACATCATC |
|  | SeLTNAT-gF1 | TAATACGACTCACTATAGCACGAAACGTAGCACGCG |
|  | SeLTNAT-gR1 | TTCTAGCTCTAAAACCGCGTGCTACGTTTCGTGC |
|  | SeLTNAT-gF2 | TAATACGACTCACTATAGGAGATGAGGCCTGGCCAGG |
|  | SeLTNAT-gR2 | TTCTAGCTCTAAAACCCTGGCCAGGCCTCATCTCC |

**Table S2** G0 mutagenesis efficiency induced by CRISPR/Cas9

| Target | Cas9/sgRNA concentration (ng/µL) | Injected embryos | Hatched larvae (%) | Pupae (%) | G0 mutation efficiency (%) |
| --- | --- | --- | --- | --- | --- |
| *LOC118263478* | 300/150 | ~200 | 30 (15.0%) | 24 (80.0%) | 83.3% (20/24) |
| *LOC118262942* | 300/150 | ~200 | 45 (22.5%) | 32 (71.1%) | 75.0% (18/24) |
| *LOC118264937* | 300/150 | ~400 | 20 (5%) | 10 (50%) | 90.0% (9/10) |
| *LOC118279045* | 300/150 | ~200 | 118 (59.0%) | 97 (82.2%) | 41.7% (10/24) |
| *LOC118279767* | 300/150 | ~200 | 121 (60.5%) | 100 (82.6%) | 50% (12/24) |
| *CsLTNAT* | 300/150 | ~800 | 122 (15.3%) | 85 (69.7%) | 64.7% (22♂/34♂) |
| *SeLTNAT* | 300/150 | ~400 | 229 (57.3%) | 150 (65.5%) | 72.9% (35♂/48♂) |

**Table S3** The species names and corresponding GenBank accession numbers for LTNAT homologs.

| Species name | GenBank accession numbers |
| --- | --- |
| *Plutella xylostella* | CAG9135299.1 |
| *Parnassius apollo* | CAG5041126.1 |
| *Parnassius Mnemosyne* | CAK1577929.1 |
| *Iphiclides podalirius* | CAH2063921.1 |
| *Battus philenor* | XP_068628014.1 |
| *Papilio Xuthus* | XP_013161602.1 |
| *Papilio machaon* | XP_014359790.2 |
| *Achroia grisella* | XP_059061171.1 |
| *Galleria mellonella* | XP_052754169.1 |
| *Amyelois transitella* | XP_013196938.2 |
| *Plodia interpunctella* | XP_053599548.1 |
| *Manduca sexta* | XP_030022837.1 |
| *Bombyx mandarina* | XP_028039519.1 |
| *Bombyx mori* | XP_004926383.1 |
| *Hyposmocoma kahamanoa* | XP_026326745.1 |
| *Pectinophora gossypiella* | XP_049865461.1 |
| *Tuta absoluta* | KAJ2938798.1 |
| *Phthorimaea operculella* | KAI5643568.1 |
| *Dendrolimus kikuchii* | KAJ0179095.1 |
| *Arctia plantaginis* | CAB3241331.1 |
| *Helicoverpa armigera* | PZC80074.1 |
| *Helicoverpa zea* | XP_047023652.1 |
| *Chrysodeixis includens* | CAH0629165.1 |
| *Trichoplusia ni* | XP_026745138.1 |
| *Mythimna separata* | KAJ8724723 |
| *Mythimna loreyi* | KAJ8726717.1 |
| *Spodoptera exigua* | CAH0668655.1 |
| *Spodoptera littoralis* | CAB3505486.1 |
| *Spodoptera litura* | XP_022829456.1 |
| *Spodoptera frugiperda* | XP_035431393.1 |
| *Diatraea saccharalis* | CAG9791702.1 |
| *Chilo suppressalis* | CAH0663851.1 |
| *Loxostege sticticalis* | KAL0839213.1 |
| *Ostrinia nubilalis* | XP_063823772.1 |
| *Ostrinia furnacalis* | XP_028170466.1 |
| *Choristoneura fumiferana* | KAI8439852.1 |
| *Cydia fagiglandana* | XP_063380444.1 |
| *Cydia splendana* | XP_063635538.1 |
| *Cydia strobilella* | XP_063530697.1 |
| *Cydia amplana* | XP_063375072.1 |
| *Cydia pomonella* | XP_061706668.1 |
| *Leguminivora glycinivorella* | XP_047989520.1 |
| *Aricia agestis* | XP_041972762.1 |
| *Leptidea sinapis* | XP_050681745.1 |
| *Zerene cesonia* | XP_038213561.1 |
| *Colias croceus* | XP_045492240.1 |
| *Colias eurytheme* | CAG4983430.1 |
| *Leptosia nina* | CAK1551502.1 |
| *Pieris napi* | XP_047519795.1 |
| *Pieris macdunnoughi* | CAF4948819.1 |
| *Pieris brassicae* | XP_045519178.1 |
| *Pieris rapae* | XP_022130974.2 |
| *Danaus chrysippus* | CAG9562860.1 |
| *Danaus Plexippus* | XP_032512336.2 |
| *Pararge aegeria* | XP_039745996.1 |
| *Bicyclus anynana* | XP_023947266.2 |
| *Maniola hyperantus* | XP_034825048.1 |
| *Maniola jurtina* | XP_045764091.1 |
| *Brenthis ino* | CAH0717513.1 |
| *Melitaea cinxia* | XP_045459922.1 |
| *Euphydryas editha* | CAH2091579.1 |
| *Nymphalis io* | XP_050362501.1 |
| *Vanessa cardui* | XP_046979045.1 |
| *Vanessa Atalanta* | XP_047545837.1 |
| *Vanessa tameamea* | XP_026491046.2 |
